# Genome-Wide Association Studies in Dogs and Humans Identify *ADAMTS20* as a Risk Variant for Cleft Lip and Palate

**DOI:** 10.1371/journal.pgen.1005059

**Published:** 2015-03-23

**Authors:** Zena T. Wolf, Harrison A. Brand, John R. Shaffer, Elizabeth J. Leslie, Boaz Arzi, Cali E. Willet, Timothy C. Cox, Toby McHenry, Nicole Narayan, Eleanor Feingold, Xioajing Wang, Saundra Sliskovic, Nili Karmi, Noa Safra, Carla Sanchez, Frederic W. B. Deleyiannis, Jeffrey C. Murray, Claire M. Wade, Mary L. Marazita, Danika L. Bannasch

**Affiliations:** 1 Department of Population Health and Reproduction, School of Veterinary Medicine University of California, Davis, Davis, California, United States of America; 2 Center for Craniofacial and Dental Genetics, Department of Oral Biology, University of Pittsburgh School of Dental Medicine, Pittsburgh, Pennsylvania, United States of America; 3 Department of Human Genetics, University of Pittsburgh Graduate School of Public Health, Pittsburgh, Pennsylvania, United States of America; 4 Department of Surgical and Radiological Sciences, School of Veterinary Medicine, University of California, Davis, Davis, California, United States of America; 5 Faculty of Veterinary Science, University of Sydney, Sydney, New South Wales, Australia; 6 Department of Pediatrics (Division of Craniofacial Medicine), University of Washington, Seattle, Washington, United States of America; 7 Center for Developmental Biology and Regenerative Medicine, Seattle Children’s Research Institute, Seattle, Washington, United States of America; 8 Department of Anatomy & Developmental Biology, Monash University, Clayton, Victoria, Australia; 9 Department of Medical Microbiology and Immunology, School of Medicine, University of California, Davis, Davis, California, United States of America; 10 Department of Surgery, Plastic and Reconstructive Surgery, University of Colorado School of Medicine, Aurora, Colorado, United States of America; 11 Division of Neonatology, Department of Pediatrics, University of Iowa, Iowa City, Iowa, United States of America; 12 Clinical and Translational Science and Department of Psychiatry, University of Pittsburgh School of Medicine, Pittsburgh, Pennsylvania, United States of America; University of Bern, SWITZERLAND

## Abstract

Cleft lip with or without cleft palate (CL/P) is the most commonly occurring craniofacial birth defect. We provide insight into the genetic etiology of this birth defect by performing genome-wide association studies in two species: dogs and humans. In the dog, a genome-wide association study of 7 CL/P cases and 112 controls from the Nova Scotia Duck Tolling Retriever (NSDTR) breed identified a significantly associated region on canine chromosome 27 (unadjusted p=1.1 x 10^-13^; adjusted p= 2.2 x 10^-3^). Further analysis in NSDTR families and additional full sibling cases identified a 1.44 Mb homozygous haplotype (chromosome 27: 9.29 – 10.73 Mb) segregating with a more complex phenotype of cleft lip, cleft palate, and syndactyly (CLPS) in 13 cases. Whole-genome sequencing of 3 CLPS cases and 4 controls at 15X coverage led to the discovery of a frameshift mutation within *ADAMTS20* (c.1360_1361delAA (p.Lys453Ilefs*3)), which segregated concordant with the phenotype. In a parallel study in humans, a family-based association analysis (DFAM) of 125 CL/P cases, 420 unaffected relatives, and 392 controls from a Guatemalan cohort, identified a suggestive association (rs10785430; p =2.67 x 10^-6^) with the same gene, *ADAMTS20*. Sequencing of cases from the Guatemalan cohort was unable to identify a causative mutation within the coding region of *ADAMTS20*, but four coding variants were found in additional cases of CL/P. In summary, this study provides genetic evidence for a role of *ADAMTS20* in CL/P development in dogs and as a candidate gene for CL/P development in humans.

## Author Summary

Cleft lip with or without cleft palate (CL/P) is a commonly occurring birth defect that can lead to a lifetime of complications in affected children. To better understand the genetic cause of these disorders, we investigated CL/P in both dogs and humans. Genome-wide association studies in both species independently identify *ADAMTS20* as a candidate gene for CL/P development. In dogs, a deletion within a functional domain of *ADAMTS20* is responsible for CL/P in the Nova Scotia Duck Tolling Retriever dog breed. In humans, an associated region containing the same gene, *ADAMTS20*, was identified in a study population of native Guatemalans. Subsequent sequencing in humans was unable to identify a causative mutation within the coding region of *ADAMTS20* in the Guatemalan cohort; however, sequencing of *ADAMTS20* in additional cases with CL/P identified four novel coding variants. This work provides genetic evidence for a role for *ADAMTS20* in CL/P development in both dogs and humans.

## Introduction

Nonsyndromic orofacial clefts, notably cleft lip (CL) with or without cleft palate (CL/P) and isolated cleft palate (CP), are the most common craniofacial birth defects in humans and represent a substantial personal and societal burden. Clefts affect approximately 1 in 700 individuals[1], with a lifetime cost of treatment in the U.S.A. estimated at $200,000[2–5]. Rates vary dramatically depending on population, with higher rates of CL/P found in Asians, South Americans, and American Indians compared with Caucasians [6], while populations of African descent are the least often affected[7]. Interestingly, the CL/P birth prevalence differs between genders (males more often affected), but the CP prevalence does not[6]. Although clefts can be surgically repaired, affected individuals often undergo multiple craniofacial and dental surgeries, as well as speech, hearing, and orthodontic therapies. Furthermore, individuals born with an orofacial cleft have increased incidence of mental health problems, higher mortality rates through all stages of life[2,8], and a higher risk of various cancer types (including breast, brain, and colon cancers) that extends to their family members[9–12].

Orofacial clefts are complex birth defects resulting from genetic variations, environmental exposures, and their interactions[3]. Before the advent of genome-wide approaches, evaluation of candidate genes revealed at best modest associations with a number of genes[13,14]; further, despite exhaustive mutation screens, coding mutations were found in less than 10% of study participants, leaving a large portion of the genetic etiology unexplained[15–18]. Genome-wide studies, using both linkage and association methods[18], have identified a number of different genes and genomic regions likely to contribute to the risk of orofacial clefts; both rare and common variants have been implicated in studies of Caucasian and Asian populations[16–18] with distinct SNPs associated in each ethnicity.

In addition to human genetic studies, a variety of model organisms have been utilized to further understand the development of orofacial clefts. A number of mammalian species exhibit orofacial clefting, with mice being the most often studied; however, mouse models often exhibit CP alone and very rarely display CL/P. In contrast, the domestic dog has spontaneous and naturally occurring CL/P with a recessive mode of inheritance documented in several breeds[19–21]. Contributing further to their usefulness as a model organism, common breeding practices have created genetically isolated populations (dog breeds) that result in few haplotypes and extensive linkage disequilibrium[22,23].

Even within closed breeding populations, genetic heterogeneity of orofacial clefts is present within the Nova Scotia Duck Tolling Retriever (NSDTR) dog breed. We previously identified a *DLX6* LINE-1 insertion as a cause of cleft palate and mandibular abnormalities in a subset of NSDTRs with orofacial clefts[24]. The *DLX6* LINE-1 insertion failed to explain a series of cases with cleft lip, indicating molecular and phenotypic heterogeneity of orofacial clefting within the breed.

Here, we present two parallel genome-wide association studies (GWAS) in the domestic dog and humans. Both studies provide evidence for a role of the same gene, *ADAMTS20*, in CL/P development. A GWAS investigating the cause of cleft lip within a small cohort of NSDTRs identified an associated interval on canine chromosome 27 that segregates with a more complex phenotype of CL/P and syndactyly. Whole-genome sequencing of these dogs led to the identification of a frameshift mutation within *ADAMTS20*. A GWAS within a cohort of native Guatemalans with CL/P identified a suggestive association with an interval encompassing *ADAMTS20*. Sanger sequencing of *ADAMTS20* within CL/P cases from the native Guatemalan cohort and additional cases, identified four novel risk variants for CL/P in humans.

## Results

### Genome-Wide Association of NSDTRs with CL/P

**Fig 1:**
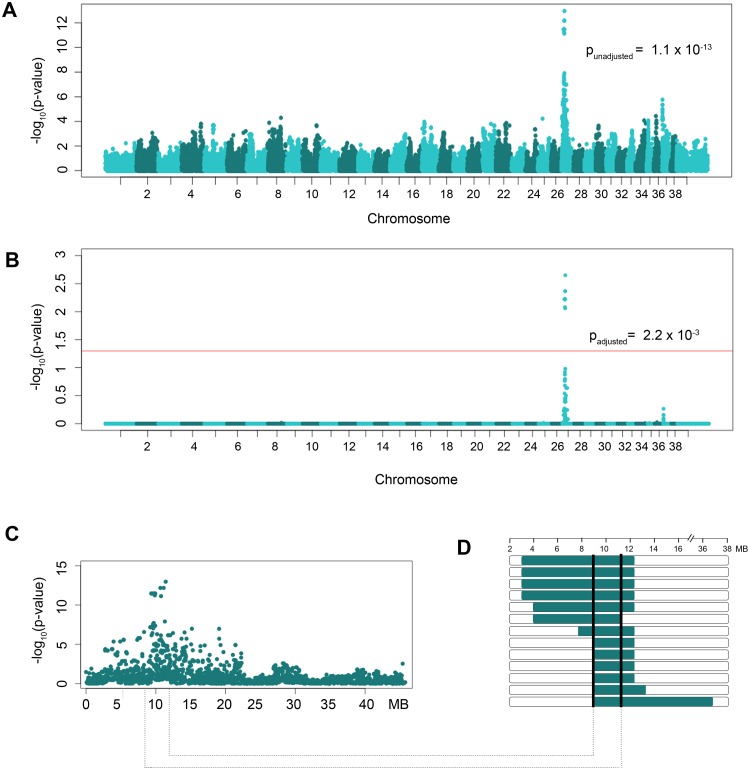
GWAS results of NSDTRs with CL/P. A. Manhattan plot of—log_10_-transformed p-values by chromosome. The most significant SNP (CFA27: 11419150) has an unadjusted p-value = 1.1 x 10^-13^. B. Manhattan plot of empirical p-values adjusted for multiple comparisons via 100,000 permutations. The red horizontal line represents a p-value ≤ 0.05 or genome-wide significance. Genome-wide significance was observed for 20 SNPs on CFA27 (lowest adjusted p-value = 2.2x10^-3^). C. Plot of the unadjusted p-values by Mb on CFA27 depicting the associated region. D. Haplotypes observed in the 13 NSDTR cases that were homozygous throughout the associated interval. Horizontal bars represent NSDTRs cases with the homozygous haplotype, with runs of homozygosity in green. The critical interval is defined by the shared homozygous haplotype denoted by the black bars (CFA27: 9.29–10.73 Mb).

To identify loci associated with CL/P in dogs, an allelic genome-wide association was performed using 7 CL/P case dogs and 112 control dogs from within the NSDTR breed. After quality control, association analysis of 110,021 remaining SNPs identified a highly associated region on canine chromosome 27 (unadjusted p-value of 1.1 x 10^-13^, CFA27: 11419150) (Fig. 1A). A genomic inflation factor (λ) of 1.18 was observed, however, the significance of the positive association and lack of any other associated chromosomal regions led to the investigation of the associated region without further correction for population stratification. After 100,000 permutations to correct for multiple tests, the adjusted empirical p-value was 2.2 x 10^-3^ with 20 SNPs reaching genome-wide significance (i.e., adjusted p-value ≤ 0.05; Fig. 1B). Underlying this evidence of association was a 2.88 Mb homozygous haplotype spanning from CFA27: 9.29–12.16 Mb in 6 cases used in the GWAS when compared to the controls (Fig. 1C). This homozygous haplotype was not identified in one of the cases with CL/P nor in any of the 112 controls.

### Homozygosity Mapping and Segregation Analysis of the Associated Interval

Homozygosity mapping of the associated interval was performed in the six full sibling cases not included in the GWAS. These six full sibling cases were excluded from the GWAS analysis in order to reduce possible population stratification. All six cases were found to be homozygous throughout the associated region. Previous studies investigating cleft palate within the NSDTR breed identified two unexplained cases of cleft palate[24]. Homozygosity mapping was also performed in these two unexplained cases and the homozygous haplotype was identified in one of the two CP NSDTRs. Closer inspection of the CP NSDTR with the homozygous haplotype revealed bilateral clefts of the lower alar nasal folds. In summary, a homozygous region concordant with the CFA27-associated interval was identified in 13 of 15 total NSDTRs with orofacial clefts (Table 1). Recombination breakpoints in the 13 dogs reduced the critical interval from 2.88 Mb to 1.44 Mb (CFA27: 9.29–10.73 Mb; Fig. 1D).

**Table 1:** Summary of NSDTRs with the associated homozygous haplotype on CFA27.

Cases of orofacial clefts	Array genotyping	Included in GWAS analysis	Homozygous haplotype
13 CL/P	13	7	12
2 CP*	2	0	1
Total with the homozygous haplotype			13

* NSDTRs from [24]

Segregation analysis of the associated haplotype was performed in unaffected family members with enough available DNA (parents n = 6; littermates n = 6). None of the 12 unaffected family members were homozygous throughout the associated interval and the parents were all heterozygous, suggesting a recessive mode of inheritance.

### Phenotypic Spectrum of CLPS in NSDTRs

**Fig 2:**
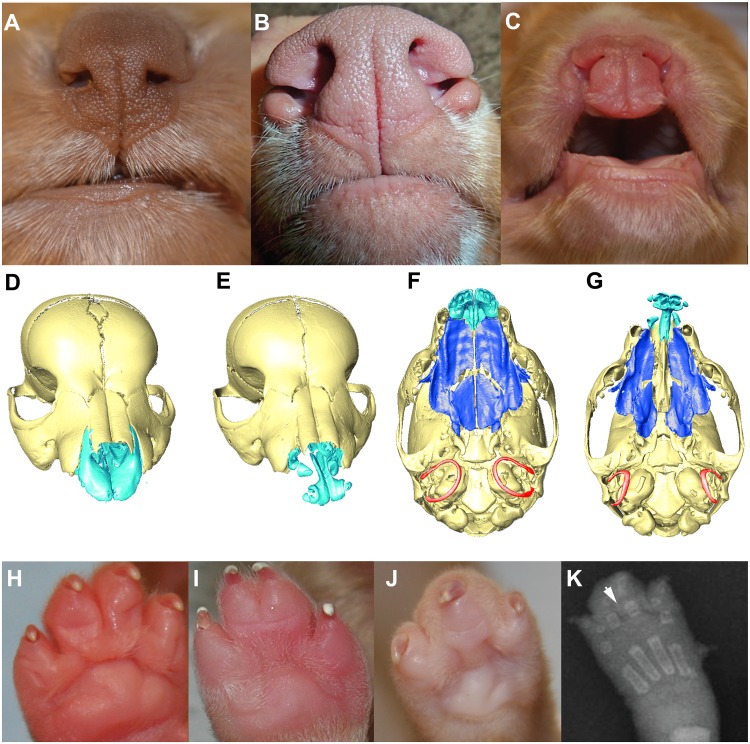
Phenotypic spectrum of CLPS in NSDTRs. A-C. Rostral view of the nose and lip. A. Wild type. B. Cleft of the lower alar nasal folds. C. Bilateral complete primary palatal cleft. D-G. 3D reconstruction of microCT imaging of puppy skulls with the mandibles digitally removed. The maxillary and palatine bones that make up the secondary palate are depicted in dark blue and the incisive bone of the primary palate is depicted in light blue. D & E. Rostral view of wild type and affected individual, respectively, showing complete bilateral cleft lip (E). F & G. Ventral view of wild type and affected individual, respectively, showing complete bilateral cleft lip, secondary palate cleft, and laterally placed tympanic rings (depicted in red). H-K.Ventral view of the paws depicting variation in syndactyly phenotypes in NSDTRs. H. Wild type. I. Incomplete syndactyly, i.e. only partial soft tissue fusion of digits three and four. J. Complete syndactyly, i.e. complete soft tissue fusion of digits three and four. K. Dorsoplantar radiograph of NSDTR right forepaw with complete syndactyly. Arrowhead points to the interdigital space demonstrating only soft tissue involvement.

The phenotypes observed in the 13 dogs with the homozygous haplotype on CFA27 include a range of clefting phenotypes including bilateral clefts of the lower alar nasal folds (n = 1), bilateral clefts of the lower alar nasal folds and CP (n = 6), a right unilateral complete CL and CP (n = 1), and bilateral complete CL and CP (n = 5) (Fig. 2A-C). To identify additional craniofacial defects, micro computed tomography (microCT) analysis was performed on two severely affected NSDTR cases with bilateral complete CL and CP and three NSDTR controls. MicroCT findings were consistent with the presence of bone and soft tissue clefts of the primary and the secondary palate (Fig. 2D-G). In both affected individuals, bilateral dentoalveolar clefting was evident between the incisors and canine teeth. This clefting was associated with a complete absence of ossification of the dorsal and lateral aspects of the premaxilla, which would normally form a suture with the maxillae and rostral aspect of the nasal bones. The ventromedial component of the premaxilla, from which the incisors arise, appeared largely normal. However, in contrast, to the controls in which the six incisors are evident in the premaxilla, the affected individuals presented with only four incisors in the remnant premaxillary bone. The lateral-most incisors were not integrated in bone and were relatively poorly formed and abnormally oriented in the cleft individuals. Interestingly, the nasal bones were normal in appearance and length. Compared to controls, the rostral portion of each maxillae was hypoplastic (S1 Fig and S2 Fig.). In one of the two individuals, a cleft of the secondary palate extending the full length of maxillary and palatine bones was observed, resulting in free communication between the nasal cavity and oral cavity. In the second individual, one palatal shelf had extended to the midline and had approximated with the anterior nasal septum while the other showed little lateral outgrowth, although the palatal rugae were still evident on both shelves. This asymmetry in shelf growth was mirrored by the asymmetric palatal bone growth. In contrast to the palate, the basisphenoid and basioccipital bones of the cranial base were similar in appearance to that of controls. Of note, however, both affected individuals had smaller (average ~20% smaller relative to skull length) and more laterally positioned tympanic rings compared to the unaffected individuals (Fig. 2FG). In one individual, the tympanic rings were also slightly dimorphic. In one individual there was notable asymmetry in length of one of the component hyoid bones. The mandible, although largely normal, had a slightly more narrow appearance and slightly delayed ossification around the mandibular canines and incisors. In both cleft individuals the forehead was slightly more tapered.

In addition to CL/P, simple complete and/or partial syndactyly of the third and fourth digits was observed in 10 of the 13 dogs (Fig. 2H-J). It is unknown whether or not the 3 remaining dogs had syndactyly. Simple syndactyly demonstrating only soft tissue involvement was observed on radiographs of a paw with complete syndactyly (Fig. 2K). We designate this phenotype cleft lip, palate, and syndactyly (CLPS). The observed phenotypic spectrum of CLPS cases is summarized in Table 2.

**Table 2:** Phenotypic spectrum observed in CLPS NSDTRs.

Type of Cleft	Syndactyly					
	All Complete	Complete rear; Incomplete front	Incomplete Rear Only	Complete Rear Only	Unknown	Total
Bilateral complete cleft lip and cleft palate	2	1			2	5
Unilateral cleft lip and cleft palate					1	1
Bilateral cleft of the lower alar nasal folds				1		1
Bilateral cleft of the lower alar nasal folds and cleft palate	2	2	2			6
Total	4	3	2	1	3	13

### Whole-Genome Sequencing

To identify variants within the critical interval, whole-genome sequencing was performed on three CLPS NSDTR cases and four control NSDTRs that were homozygous wild type throughout the associated interval and unaffected with CLPS. Within the associated interval (CFA27: 9.29–10.73 Mb) there were 14,167 SNP and in/del variants when compared to the CanFam 3.1 boxer reference genome [22]. Based on the homozygous haplotype observed in the cases, we hypothesized a recessive mode of inheritance and excluded variants that did not segregate with the phenotype. From the set of homozygous variants in the CLPS cases, we excluded variants that were also homozygous in at least one control dog. This reduced the number of variants segregating with the phenotype to 142 (Table 3), when compared to a reference set of 26 control dogs. Breeds of all dogs with whole genome sequence are summarized in S1 Table.

**Table 3:** Summary of variants within the 1.44 Mb critical interval on canine chromosome 27.

Variant Type	Number of Variants
Total SNP and Indel variants in 1.44 Mb	14167
Variants that segregate across 7 NSDTR genomes	582
Variants that segregate across all 33 dog genomes	142

Based on alignments to CanFam 3.1 Boxer reference sequence. Only variants that are homozygous in all cases and not homozygous in any controls were considered to segregate and could be excluded. Variants were filtered against a reference set of 26 control dogs with whole genome sequencing data.

**Fig 3:**
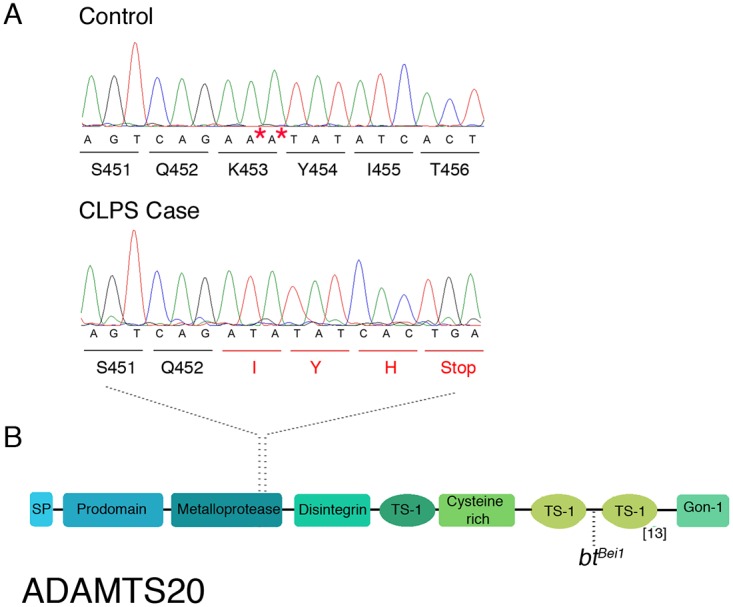
Characterization of *ADAMTS20* mutation in NSDTRs. A. Chromatograms of *ADAMTS20* cDNA sequence from embryo control and NSDTR case depicting the AA deletion and predicted premature stop codon. * denotes the deleted nucleotides in the 13 NSDTR cases. B. Schematic functional domain organization of ADAMTS20. SP- signal peptide TS- Thrombospondin type 1 motif[26]. *bt* ^*Bei1—*^reflects the location of point mutation responsible for the *bt* ^*Bei1*^allele.

Of these 142 variants, only two were predicted to affect the coding region of genes within the critical interval (S2 Table) [25]. One synonymous coding variant (*PUS7L* c.278A>G (*p*. *(=))*) was identified as homozygous in cases and heterozygous in 10 control dogs (1 NSDTR, 5 Dalmatians, 1 Kelpie, 1 Bearded Collie, 2 Weimaraners). Due to the severe nature of the CLPS phenotype, we hypothesized that the causative allele would occur at a low frequency across all dog breeds. Because approximately one third of the randomly sampled control dogs were heterozygous for *PUS7L* c.278A>G (*p*. *(=)*), we concluded that this variant was unlikely to be causal for the CLPS phenotype. The second variant, *ADAMTS20* c.1360_1361delAA (p.Lys453Ilefs *3), was predicted to be a frameshift mutation in the metalloprotease domain resulting in premature truncation of 1461 amino acids from the 1916 amino acid protein (Fig. 3AB) [26]. It was not observed in any of the 30 control dogs.

### Mutation Validation

To confirm the *ADAMTS20* c.1360_1361delAA (p.Lys453Ilefs*3) frameshift mutation, Sanger sequencing was performed in cDNA from a CLPS NSDTR case and embryo control (Fig. 3A). In addition to the deletion, seven SNPs were identified in the CLPS NSDTR case and embryo control when compared to CanFam 3.1 Boxer reference sequence (Table 4)[22]. Six of the 7 SNPs did not segregate with the phenotype. *ADAMTS20* c.682C >T (p.Val228Leu) segregated within the CLPS NSDTR case, embryo control, and reference sequence, but is a known SNP previously identified in wolves and 15 other dog breeds[27]. Further investigation of this SNP in the 8 NSDTRs with available whole-genome sequence also confirmed that this SNP did not segregate with the phenotype: 1 NSDTR was homozygous for the boxer reference allele, 3 NSDTRs were heterozygous, and 3 NSDTRs were homozygous for the alternate allele (3 cases, 1 control).

**Table 4:** SNPs identified within the canine *ADAMTS20* coding region.

Genomic Location	Boxer Reference	Embryo Control	NSDTR Case	SNP Name	SNP Effect
10493347	G	C	C	no known SNP	c.302G>C (p.Gly101Arg)
10528180	C	C	T	SNP2857855	c.682C>T (p.Val228Leu)
10542397	C	T	T	SNP2857888	c.997C>T (*p*. *=*)
10564700	C	T	T	SNP2857941	c.1860C>T *(p*. *=*)
10624210	C	G	G	SNP2858090	c.4660C>G (p.Gln1554Glu)
10624314	T	C	C	SNP2858091	c.4764T>C (*p*. *=*)
10626762	T	G	G	no known SNP	c.4834T>G (p.Leu1612Val)

Genomic locations are based on the CanFam3.1 assembly and refer to chromosome 27 base pair locations. The SNPs identified are based on UCSC Genome Browser CanFam 3.1 Broad Improved Canine Annotation v1 SNPs track[27].

We hypothesize that the predicted premature stop codon in *ADAMTS20* c.1360_1361delAA (p.Lys453Ilefs*3) results in decreased expression levels when compared to wild type. Quantitative real time PCR analysis was performed on cDNA from heart tissue of CLPS NSDTRs and compared to cDNA from heart tissue of unaffected NSDTRs that were homozygous wild type for the deletion. REST analysis indicated that the *ADAMTS20* transcript in CLPS NSDTRs was significantly down regulated by a mean factor of 0.549 (p = 0.005) in CLPS cases when compared to controls[28].

### CLPS Allele Frequency

**Fig 4:**
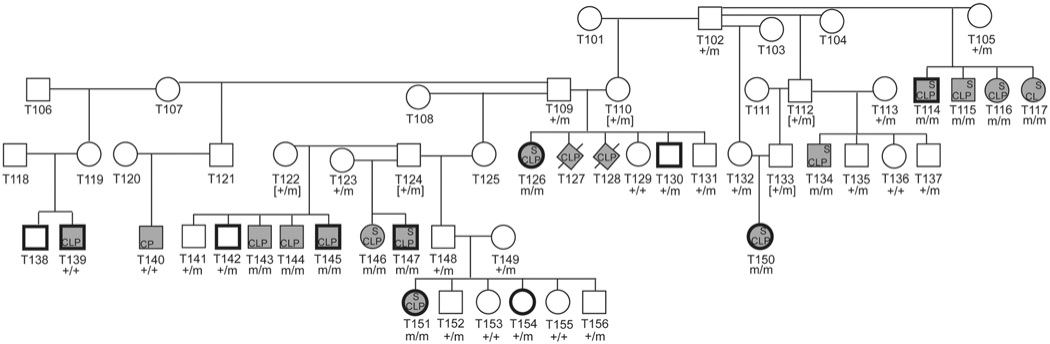
Pedigree of NSDTR families depicting segregation of the phenotype with the *ADAMTS20* deletion. Filled symbols represent NSDTR cases. Bold symbols represent NSDTRs that were included in the GWAS. Not all relationships are included. Abbreviations: CLP = cleft lip and palate, CP = cleft palate, S = syndactyly, + = wild type allele, m = *ADAMTS20* two base pair deletion, and [] = DNA was not available and genotypes were inferred.

Genotyping was performed in available parents (n = 8), littermates (n = 13), and all NSDTR cases (n = 13 CLPS; 2 unexplained) (Fig. 4). All 13 CLPS cases were homozygous for the deletion. The two unexplained NSDTRs cases without the associated haplotype were homozygous for the wild type allele. We genotyped 97 unrelated control NSDTRs and identified this deletion in the heterozygous form in 3 control dogs. To determine if this allele is found in other breeds, we genotyped 53 dogs with orofacial clefts from 25 breeds and 288 unaffected dogs from more than 70 breeds (S3 Table). All genotyped individuals were homozygous for the wild type allele, suggesting this *ADAMTS20* deletion is private to the NSDTR breed.

Our previous work in dogs identified a *DLX6* LINE-1 insertion responsible for cleft palate and mandibular abnormalities in a subset of NSDTRs [24]. The *ADAMTS20* c.1360_1361delAA (p.Lys453Ilefs*3) deletion was also genotyped in these cases (n = 19). Two of the cases were heterozygous, while the remaining 17 dogs were homozygous wild type for the *ADAMTS20* c.1360_1361delAA (p.Lys453Ilefs*3) mutation. Of all control NSDTRs, including relatives, genotyped for both mutations (n = 99), five were heterozygous for both mutations and were phenotypically normal.

### Genome-Wide Allelic Association in Native Guatemalans

**Fig 5:**
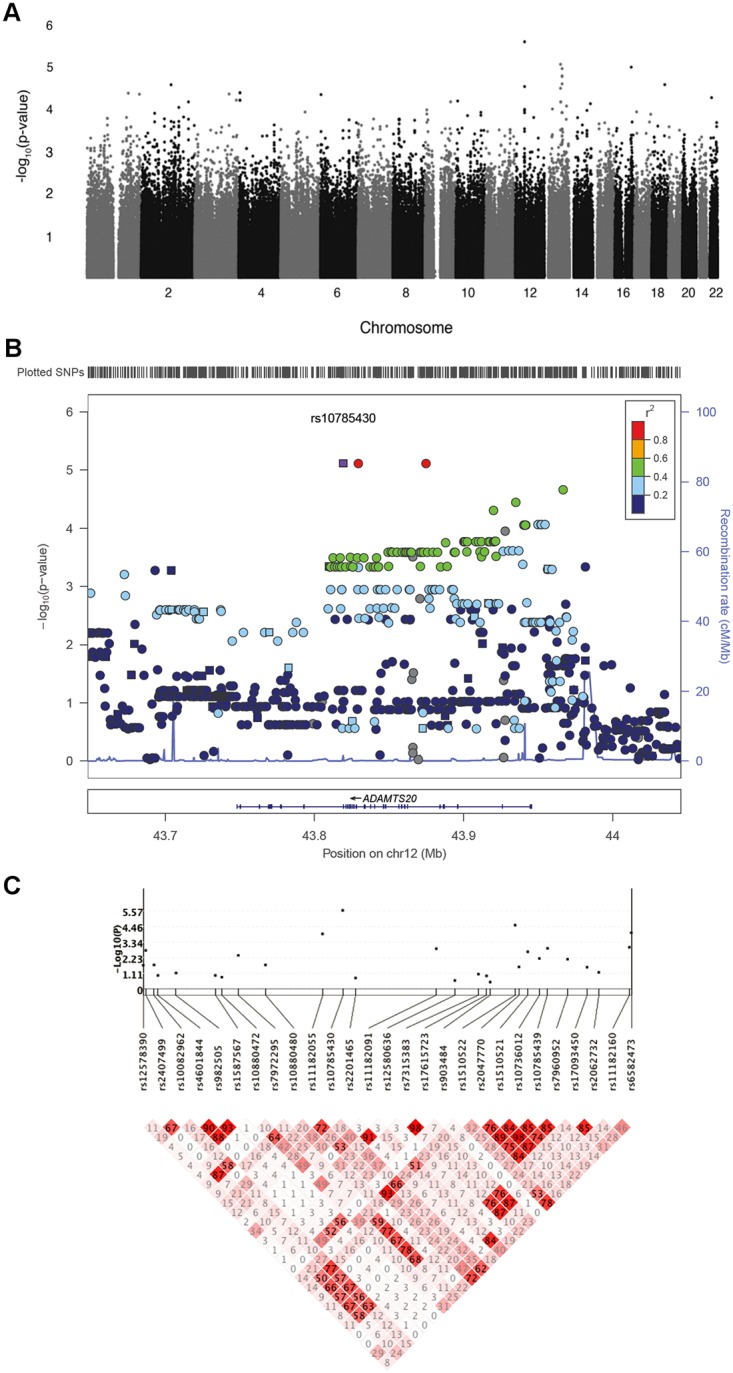
Genetic association results for CL/P in Guatemalans. A. Manhattan plot showing-log_10_-transformed p-values from genome-wide DFAM analysis of CL/P. Analysis included 125 affected and 812 unaffected participants. B. LocusZoom plot showing—log_10_-transformed p-values (left y-axis) for genotyped (squares) and imputed (circles) SNPs in and near *ADAMTS20* on chromosome 12. The recombination overlay (blue line, right y-axis) indicates the boundaries of the LD-block containing *ADAMTS20*. The density of SNPs in this region is indicated by the rug plot (top). C. KGG.v2 gene plot showing association and linkage disequilibrium (LD; r^2^) of genotyped SNPs in *ADAMTS20*.

To investigate the cause of CL/P in humans, a DFAM allelic association[29] analysis was performed in a Guatemalan study population of 125 nonsyndromic CL/P cases, 420 unaffected relatives (545 total from case families), plus 392 controls with no family history of orofacial clefts (Fig. 5A). No p-value reached a conservative Bonferroni corrected p-value of less than 1.1 x 10^-7^ (alpha = 0.05), but several SNPs had p-values suggestive of association (p-values ~10^-6^). Table 5 shows the top 5 CL/P associated SNPs according to the DFAM analysis within the Guatemalan cohort, as well as the corresponding TDT, sib-TDT, and logistic regression p-values. S4 Table lists all SNPs with DFAM p-value less than 0.0001. The QQ plot of the allelic GWAS results shows no evidence of genomic inflation in this family based study (S3A Fig.).

**Table 5:** Single SNP analysis of human GWAS data.

CHR	SNP	GENE	MAF*	P-value (Rank)			
				DFAM [n = 937]	TDT [n = 65]	Sib-TDT [n = 49]	Case [n = 113] Control [n = 241]
12	rs10785430	*ADAMTS20*	0.44	2.69E-6 (1)	1.83E-4 (71)	3.81E-4 (62)	0.007 (9997)
13	rs9530247	*KLF12*	0.47	9.15E-6 (2)	1.27E-5 (5)	0.03 (14072)	0.07 (62887)
16	rs3743613	*BCAR1*	0.49	1.07E-5 (3)	5.54E-5 (24)	0.01 (4477)	0.59 (306308)
13	rs7327912	*(none)* ^*#*^	0.29	1.16E-5 (4)	2.34E-5 (7)	0.01 (4829)	0.06 (53228)
13	rs17073177	*(none)*	0.29	1.72E-5 (5)	3.65E-5 (17)	0.01 (4829)	0.06 (54290)

*Calculated from unaffected founders only

^#^ 400kb downstream from *SPRY2*

The strongest association observed was for rs10785430 (DFAM p-value = 2.69 x 10^-6^) on chromosome 12, which mapped to an intron in *ADAMTS20*. This SNP also showed significant associations (p<0.05) using the TDT (p = 1.8 x 10^-4^), sib-TDT (p = 3.8 x 10^-4^), and case-control (p = 0.007) analyses. To further explore this region, we imputed unobserved SNPs on chromosome 12 using the 1000 Genomes reference sample (http://browser.1000genomes.org). Based on SNPs in high LD with rs10785430, association tests revealed a 157kb region of association (from rs11182055 to rs9988939) yielding p-values less than 1x10^-4^ (Fig. 5B).

We next performed a gene-level analysis with DFAM on 17,578 genes. The QQ plot of gene p-values revealed no significant deviations from expected (S3B Fig.) indicating that the VEGAS method adequately controlled for LD (shown for *ADAMTS20* in Fig. 5C). The top 5 associated genes derived from the Guatemalan GWAS using the VEGAS method are summarized in Table 6. *ADAMTS20* had the lowest gene-wise p-value (p = 5.3x 10^-5^), in agreement with the single SNP results.

**Table 6:** VEGAS results using DFAM p-values from human data.

Chr	Gene	^#^ SNPs	Start	Stop	P-value*	Top SNP	SNP P-value
12	*ADAMTS20*	27	42034278	42231991	5.3E-5	rs10785430	2.7E-6
16	*BCAR1*	13	73820428	73843004	1.1E-4	rs3743613	1.0E-5
8	*FZD3*	14	28407691	28477880	2.1E-4	rs2241802	2.0E-4
8	*FBXO16*	11	28341847	28403703	2.1E-4	rs7001034	2.6E-4
11	*SNF1LK2*	12	110978379	111102842	6.5E-4	rs510388	3.13E-4

* p-value for the gene-based analysis

^#^ p-value

Previous genome-wide studies were performed in Caucasian and Asian trios[17,18], but did not identify *ADAMTS20* as a top hit. However, several SNPs within *ADAMTS20* did show nominal significance (p<0.05) in Caucasians[17], so we performed a meta-analysis of the Guatemalan and Caucasian results for the *ADAMTS20* SNPs. Overall, smaller p-values were observed for some SNPs after the addition of the Caucasian results, but not for the most significant SNP in the Guatemalans (S4 Fig.). This suggests a distinct genetic etiology for CL/P formation in Guatemalans.

### Sequencing of *ADAMTS20* in Human CL/P Cases

We sequenced all protein coding exons of *ADAMTS20* in 20 Guatemalan CL/P cases to determine if a novel, population-specific common variant could explain the association with markers in *ADAMTS20*; however, no such variants were identified. We also sequenced 19 cases from the Philippines to explore the possibility that rare variants in *ADAMTS20* could confer risk of CL/P in other populations. Similarly, we looked for rare coding variants in our Guatemalan cases that could contribute to CL/P risk independent of the association with the common SNP rs10785430. No private variants were found in the Guatemalan cases, but three novel missense variants were found among Filipino cases (S5AB Fig.). All three novel Filipino variants were inherited from unaffected parents. Notably, two of these variants occurred on a common haplotype and were found in both affected children in the family (S5A Fig.). A summary of all of the variants found in the nonsyndromic CL/P cases is found in S5 Table. We also sequenced 44 individuals of diverse ethnicities with CL/P plus syndactyly or limb defects including amniotic bands, polydactyly, and ectrodactyly (which was motivated by the CLPS phenotype observed in dogs which includes syndactyly). From these samples, only one missense variant (chr12: g.43824214C>T (p.A1108T)) was found in an individual with CL/P, facial asymmetry, and a single transverse palmar crease of the left palm. However, this variant did not segregate with clefting in the family (S5C Fig.).

## Discussion

This study presents independent genome-wide association studies that provide evidence of the involvement of *ADAMTS20* in the development of orofacial clefts in dogs and humans. The canine study identified a 1.44 Mb region of homozygosity underlying an association on CFA27 where subsequent whole-genome sequencing identified a frameshift mutation in *ADAMTS20* that segregated with a complex phenotype of syndromic cleft lip, cleft palate, and syndactyly. The parallel human studies applied combinational-based association statistics to identify suggestive allelic (DFAM) and gene-level (VEGAS) associations with SNPs in *ADAMTS20* in a cohort of native Guatemalans with nonsyndromic CL/P. Both studies identify *ADAMTS20* as a candidate gene for CL/P development in humans.

This work describes a second causative mutation for an independently segregating locus of orofacial cleft formation within the NSDTR dog breed. CLPS is characterized by a syndromic form of cleft lip, cleft palate, and syndactyly that segregates with a recessive mode of inheritance. This is independent of the previously identified CP1 locus that is characterized by a cleft palate and shortened mandible[24]. A genetic cause has not been identified in two cases of orofacial clefts within NSDTRs, further exemplifying the genetic heterogeneity within the NSDTR that has been previously documented[24]. This heterogeneity mimics what is observed in human cleft cases and is likely indicative of what will be observed in other dog breeds.

ADAMTS20 (a disintegrin-like and metalloprotease with thrombospondin type-1 motifs) is part of a large family of secreted zinc metalloproteases sharing a similar domain organization[26] that are involved in cleaving extracellular matrix (ECM) proteins and processing procollagen[30]. ADAMTS20 cleaves the ECM proteoglycan, versican[26,31], and is involved in a variety of biological processes including promotion of melanoblast survival, palatogenesis, and interdigital web regression[31–33].

Expression studies in mouse embryos identify craniofacial expression of *Adamts20* in the first pharyngeal arch, between the medial nasal processes[34], and broad expression in the palatal mesenchyme, where it plays a role in the sculpting and extension of the palate[32]. *Adamts20* is also expressed in the developing fore- and hind limbs, the interdigital tissue, and at the medial border of the developing autopod[33,34].

In mice, mutations in *Adamts20* are best known to cause a fully penetrant recessive, ventral to dorsal white belted phenotype (*bt*)[34]. In addition, *bt* mice have a low penetrance of cleft palate (3%) and soft tissue syndactyly (18%)[32,33]. Full penetrance of cleft palate was observed in *bt* mice with additional mutations in *Adamts9 (Adamts9* ^*+/-*^;*bt/bt*)[32]. Within the NSDTRs, the CLPS deletion results in 100% penetrance of primary palate clefts. There is variation in the primary palate phenotype that ranges from clefting of the lower alar nasal folds to bilateral cleft lip. Secondary palate clefts are 92% penetrant, but also exhibit some variability in presentation. Syndactyly is likely fully penetrant as it was observed in 10 of the 13 dogs and the status of three remaining full-sibling cases is unknown. NSDTRs have minimal white spotting segregating in the breed, but none of the dogs with the associated haplotype exhibited any obvious midline white markings similar to what is observed in *bt* mice. This work complements what has been identified within the mouse by providing further evidence for role of *ADAMTS20* in cleft palate and syndactyly formation. This highlights that *ADAMTS20* should further be investigated for its role in CL/P and craniofacial development.

Previous work describing mutations in *ADAMTS20* and other ADAMTS family members may provide insight into the observed phenotype of *bt* mice and CLPS NSDTRs. ADAMTS proteins share identical N-terminal domains (e.g. metalloprotease), but the type and number of C-terminal ancillary domains vary. These ancillary domains are critical for activity, inhibition, tissue localization, and substrate specificity[35]. Work on ADAMTS13 demonstrated that point mutations do not consistently function as null alleles[36] and deletion of different ancillary domains in ADAMTS5 and ADAMTS13 resulted in mutant constructs that retained partial function depending on the specific domains that were deleted[37–40]. Point mutations have been described in four *bt* alleles (*bt*—c.1598C>T p.Pro533Leu; *bt* ^9J^—c.2451C>T p.Leu761Phe; *bt* ^*Bei1*^—c.2860C>T p.Arg954Ter; *bt* ^*Mri1*^—c.4073A>C p.His1357Pro), which are located downstream of the catalytic metalloprotease domain[31,34]. In comparison, the deletion identified in CLPS NSDTRs (c.1360_1361delAA (p.Lys453Ilefs*3)) is closer to the N terminus and within the catalytic metalloprotease domain (Fig. 3B)[31,34]. Furthermore, the allele commonly used to study the *bt* phenotype (*bt* ^*Bei1*^) is a nonsense mutation that truncates 471 amino acids or 33% of the full-length protein (Fig. 3B)[32,34]. In ADAMTS13, truncation after the spacer domain (a mutation similar to the nonsense *bt* ^*Bei1*^ allele) results in a metalloprotease that is still active[37,38]. The mutation identified in CLPS NSDTRs truncates 1461 amino acids or 76% of full-length protein and may explain the higher penetrance of craniofacial defects and syndactyly observed in CLPS NSDTRs. In summary, this mutation could be the result of a more severe hypomorph than *bt*, a null, or a species difference. It is also interesting to note that the CLPS NSDTRs do not appear to have the white spotting pattern that is characteristic of the *bt* mice. Further studies examining the activity and regulation of ADAMTS20 will be necessary to dissect the molecular impact of the CLPS mutation and to determine if it is a null allele or a hypomorph.

*ADAMTS20* transcript expression levels are observed at only 55% in CLPS NSDTRs when compared to wild type NSDTRs. Transcripts with premature stop codons often undergo degradation by nonsense mediated mRNA decay to prevent accumulation of truncated proteins[41,42], but often much lower expression levels are observed[43]. Since expression levels were analyzed in neonatal heart tissue, it is possible that a further decrease in expression levels may be observed in the appropriate tissue during the correct developmental time point.

Although no SNP reached genome-wide significance in the human GWAS, the data presents a suggestive association with a SNP within *ADAMTS20* (rs10785430). A similar phenotype observed in dogs and mice with mutations in the same gene, combined with the biological relevance of *ADAMTS20* to development of the phenotype observed in the Guatemalans, suggests that this gene should be further investigated for CL/P development in humans. These results also indicate that even suggestive loci identified in human GWAS warrant further investigation.

Sequencing of individuals with nonsyndromic CL/P from Guatemala and the Philippines did not identify any coding variants with obvious functional impact. Since the GWAS result suggests the existence of one or more common etiologic variants, it is possible that rare coding variants may yet be found in a subset of individuals with CL/P. We hypothesize that the etiologic variant(s) will be located in regulatory elements of *ADAMTS20*, perhaps located within introns. We also sequenced *ADAMTS20* in an additional cohort of individuals with syndromic CL/P who also had syndactyly or other limb defects. Although we did not find any coding variants in these individuals, *ADAMTS20* mutations causing a cleft and syndactyly syndrome may be extremely rare. Variants in and around *ADAMTS20* may also act as modifiers of the phenotype in clefting syndromes that including syndactyly as part of the phenotypic spectrum, such as Van der Woude syndrome and the ectodermal dysplasias.

In conclusion, we showed separate association studies in humans and dogs that provide evidence of association between CL/P and variants in *ADAMTS20*. This complements what is known in the mouse and suggests that *ADAMTS20* should be further investigated for its role in CL/P development. Notably, dogs have long been used as models for craniofacial surgical techniques, but this study also demonstrates that they have the potential to be relevant models for the genetics of CL/P. Here we highlighted how dogs are a genetically amenable model organism with naturally occurring cleft lip (the most common orofacial cleft type in humans) and provide a different genetic background to study mutations.

## Materials and Methods

### Ethics Statement

Subjects for this study were recruited from various sites in Guatemala as part of the Pittsburgh Oral-Facial Cleft study, a large research program investigating factors that contribute to the development of CL/P and CP. Recruitment was done in collaboration with the nonprofit organization Children of the Americas (www.childrenoftheamericas.org/).

Individuals and their family members seeking cleft lip and palate repair between 2004 and 2010 at multiple sites in Guatemala (San Juan Sacatepequez, Huehuetenango, Tiquisate, Quiche and Retalhuleu) were invited to participate in our study. This project was approved by both the University of Pittsburgh Institutional Review Board and the Oversight Ethics Committees of each of the participating hospitals; all participants gave informed, written consent in their native languages. Age appropriate assent documents were used for children between 7 and 14 years of age and informed, written consent was obtained from the child, as well as from the parents.

Collection of canine samples in this study was approved by the University of California, Davis Animal Care and Use Committee (protocol #16892).

### Canine Samples and Phenotyping

Buccal swabs, blood, or tissue samples were collected from privately owned NSDTR dogs from the following phenotypic groups: CL/P (n = 13); CP with a previously identified *DLX6* LINE-1insertion (n = 19)[24]; CP without a known causative mutation (n = 2; 1 later identified to have CL/P)[24]; healthy littermates of CL/P NSDTRs (n = 13); healthy parents of CL/P NSDTRs (n = 8); control NSDTRs (n = 205). Samples from dogs with orofacial clefts across 25 other breeds (n = 53) were also obtained from privately owned dogs. Blood samples from control dogs (n = 288) across 70 other breeds were collected from the William R. Pritchard Veterinary Medical Teaching Hospital (VMTH). Embryos were collected from pregnant bitches undergoing ovariohysterectomy at the VMTH. Embryos were staged based on measurements of crown-to-rump length and the observation of external features[44].

Gross phenotypic evaluation of the orofacial clefts were performed by a board certified veterinary dentist who is experienced in the evaluation of cleft palate. Further phenotyping was performed by high-resolution microCT imaging of two CLPS NSDTR cases and three NSDTRs controls as previously described[24]. Genomic DNA was extracted from whole blood, tissue samples, or buccal swabs using Qiagen kits (Valencia, CA).

### Genome-Wide Association

Genome-wide SNP genotyping was performed in 13 cases and 112 controls using the Illumina CanineHD BeadChip with 173,662 markers. All samples had a genotyping call rate of ≥ 90%. 62,546 SNPs were excluded due to a minor allele frequency ≤ 5% and 3,199 SNPs were excluded for a high failure rate (≥ 10%). 110,021 SNPs were used in the final analysis. Chi-square analysis was performed in PLINK[29] on one case from each litter (n = 7) and discordant sibling pairs (n = 4) when available. 100,000 permutations were performed to correct for multiple tests and the genomic inflation factor was calculated in PLINK[29]. Segregation analysis of the associated haplotype was performed in parents (n = 6) and littermates (n = 6) with enough available DNA for genotyping on the Illumina CanineHD BeadChip. Homozygosity throughout the associated interval was analyzed by visual inspection facilitated by color-coding homozygous genotypes in excel. The genotypes are available from the Dryad Digital Repository (doi:10.5061/dryad.j8r8q).

### Whole-Genome Sequencing

Seven NSDTRs were selected for whole-genome sequencing. Three CLPS NSDTR cases that were representative of the phenotypic spectrum were selected for sequencing: bilateral CL and CP with complete syndactyly of all paws, bilateral cleft of the lower alar nasal folds and CP with complete rear and incomplete front syndactyly, and bilateral cleft of the lower alar nasal folds with CP and incomplete rear syndactyly. Four control NSDTRs were selected for sequencing that were homozygous wild type throughout the associated region identified on CFA27. Two of the four NSDTR controls had normal craniofacial structures. One NSDTR control had a cleft palate and shortened mandible explained by a *DLX6* LINE-1 insertion[24] and the remaining NSDTR had a bilateral complete cleft lip with an unexplained genetic cause.

Library preparation and DNA sequencing was carried out by the Ramaciotti Centre at the University of New South Wales, Kensington. Genomic DNA was size selected for 500bp fragments and sequenced on the HiSeq 2000 sequencing platform (Illumina, San Diego, CA) according to vendor’s instructions. Paired-end reads of 101 bp were generated for each sample on a single lane of the sequencer’s flow cell, yielding between 179 and 233 million read pairs per individual. Assuming a genome size of 2.5 Gb, this data reflected raw coverage of 14.4–18.8 fold.

The canine genome sequence (Canfam3.1;[22]) was retrieved from the University of California, Santa Cruz genome browser (UCSC, http://genome.ucsc.edu) and indexed with the Burrows-Wheeler Transform Smith Waterman tool in the Burrows-Wheeler Alignment (BWA) package version 0.6.2[45]. Reads were aligned as pairs to the indexed reference genome using BWA, applying the default parameters for paired-end read alignment using this package. Alignment statistics generated using the idxstats tool within the SAMtools package version 0.1.18[46] indicated average mapped coverage ranging between 14.1 and 16.8 fold per individual. Snpsift was used to sort variants within the 1.44Mb interval by the ‘hom’ ‘any’ case and control filter options[25]. Annotation of remaining variants was performed with SnpEff using the xenoRefGene genes and gene prediction tracks annotation downloaded from the Table Browser window on the UCSC Genome Browser[25]. VCF files of the critical interval are available from the Dryad Digital Repository (doi:10.5061/dryad.j8r8q).

### cDNA Sequencing

All primers described were designed in Primer 3 (see S6 Table). Expression of *ADAMTS20* was evaluated as described previously[24]. Total RNA was isolated from tissue samples using Qiagen QIAamp Blood Mini Kit tissue protocols. RNA was synthesized into cDNA using Invitrogen Superscript III First Strand Synthesis System to RT PCR protocols. *ADAMTS20* cDNA was PCR amplified in heart tissue from one NSDTR case and a whole embryo (collected at day 30) control. Areas with high GC content were amplified using Invitrogen AccuPrime GC-Rich DNA Polymerase protocols. PCR products were sequenced on an ABI 3500 Genetic Analyzer and analyzed using Vector NTI (Informax, Frederick, MD, USA). Sequences were aligned to each other and Boxer (Can Fam 3.1) reference sequence to identify any polymorphisms[22].

### Quantitative Real-Time PCR

Primer sequences were generated using Primer3Plus (http://primer3plus.com/) (S6 Table). Semi-quantitative PCR using AmpliTaq Gold DNA Polymerase was performed to test the quality of cDNA and primers, to confirm product size, and to check for the presence of genomic DNA contamination. Real-time PCR was performed using the Rotor-Gene SYBR Green PCR Kit (QIAGEN, Valencia, CA) using a 2-step cycle protocol (35 cycles; Initial denaturation-5 minutes at 95°C; Annealing- 5 seconds at 95°C; Extension- 10 seconds at 60°C; Final Melt curve) on the Rotor Gene Q real-time PCR system. cDNA from heart tissue of 3 neonatal NSDTR controls and 3 neonatal CLPS NSDTR cases were run in triplicates with each replicate containing 4–5 ng template cDNA. All data was normalized to the housekeeping gene, *B2M*[47]. Amplification and takeoff values were analyzed and graphed by REST2009[28] to determine any significant expression differences in *ADAMTS20* transcript levels between case and control cDNA samples.

### Genotyping of Canine Samples

PCR genotyping was performed according to standard protocols[24] using a shared FAM labeled forward primer (S6 Table). GeneScan 500 ROX size standards were used and the reaction was analyzed on an ABI 3500 Genetic Analyzer. 97 unrelated control NSDTRs, 288 control dogs across 70 breeds, and 53 dogs with orofacial clefts across 25 breeds were genotyped for the deletion. All genotypes were analyzed using ABI GeneMapper software.

### Human Samples and Phenotyping

From the larger study population, 937 individuals were genotyped (see methods below): 545 from case families (125 affected with nonsyndromic CL/P and 420 unaffected relatives), plus 392 controls with no family history of orofacial clefts. From the genotyping, the population structure of the study subjects was compared to HapMap controls (see S6 Fig.). The results show some European Caucasian admixture based on HapMap controls, plus substantial overlap with the Mexican HapMap Controls. All participants self-identified as Mayan; many spoke Quichean as well as Spanish. Trained health care professionals evaluated cleft phenotypes and ruled out syndromes for each participant. Each participant also provided detailed demographics, medical history, and family history, and female participants provided a detailed pregnancy history. Blood or saliva samples were obtained for DNA extraction using Qiagen kits (Valencia, CA).

### Genome-Wide Genotyping of Human Samples

Study participants were genotyped for 620,901 markers on the Illumina Human610-Quad (Illumina Inc, San Diego, CA, USA). All individuals had a genotyping rate greater than 90% and therefore were included in the analysis. Deviations from Hardy-Weinberg equilibrium assessed in PLINK[29] found 622 markers with significant deviation from expectation in founder controls (p ≤ 1e-06). An additional 47,687 SNPs were eliminated because of high genotype failure rate (≥10%). An exclusion criterion of a minor allele frequency <5% in founders removed 105,758 SNPs. After removing non-autosomal SNPs a total of 457,969 SNPs remained for the analyses reported here. To further explore a putative association found on chromosome 12 (see results below), we imputed 270,467 SNPs on this chromosome using IMPUTE2 software[48] and the 1000 Genomes Project as the reference sample (http://browser.1000genomes.org). The genotypes and phenotypes for the Guatemalan study population are available in dbGaP (http://www.ncbi.nih.gov/gap), accession number phs000440.v1.p1.

### Allelic Association Methods

Due to the heterogeneous family structures in our Guatemalan cohort, we performed an association analysis using the DFAM test implemented in PLINK, which integrates a standard TDT, discordant sib-TDT, and Cochran-Mantel-Haenzel clustered-analysis for case-control testing[29]. Independence between each test is established by considering individuals only once in the above statistical tests. For example, participants with relevant standard TDT information were not considered in sib-TDT or Cochran-Mantel-Haenszel clustered analysis. The order of assignment begins with a standard TDT, followed by a sib-TDT, then all remaining unrelated members are considered for the case-control analysis.

To verify the top hits from the DFAM analysis we separately ran a standard TDT (-tdt option in PLINK[29,49], n = 65 trios), a sib-TDT (-dfam command in PLINK in non-founders from nuclear families with multiple siblings and at least one affected family member, n = 49 families), and a case-control test (113 randomly chosen cases and 241 controls). To maximize sample size within these tests, we investigated each using all potential participants with the understanding the derived p-values are not necessarily independent between tests. For case-control analyses, we used logistic regression under the additive genetic model where genotypes were coded by the number of minor alleles (0,1,2) in each case or control.

### Gene-Based Association Tests

In addition to the DFAM analysis, we used a multivariate analysis to combine multiple SNPs within a gene into a single statistic. Gene level analysis has the potential benefit of increased power to detect associated regions containing multiple moderate effects[50]. VEGAS is a versatile gene-based test designed to handle any type of data input as long as a p-value can be generated for each individual marker[51]. Within a gene, p-values are converted to an upper tail chi-squared statistic with one degree of freedom and then combined. An empirical null distribution is created from a Monte Carlo simulation on a multivariate normally distributed random vector with a correlation equal to those predicted from a reference population through a Cholesky decomposition matrix. The proportion of simulated test statistics exceeding the observed gene-based test statistic gives the empiric p-value. In this situation, founders from the Guatemala data set were used as a reference population since no publicly available genetic data set sufficiently matches the participants’ genetic background. Gene plots with LD diagrams were generated with Locus Zoom and KGG2.5[52,53].

### Sequencing of Human Samples

Primers covering the protein coding exons of *ADAMTS20* were designed with Primer3 (http://frodo.wi.mit.edu/primer3/). Primer sequences and annealing temperatures are available in S7 Table. PCR products were sequenced on an ABI 3730XL (Functional Biosciences, Inc., Madison, WI). Chromatograms were then transferred to a Unix workstation, base-called with PHRED (v.0.961028), assembled with PHRAP (v. 0.960731), scanned by POLYPHRED (v. 0.970312), and visualized with the CONSED program (v. 4.0). The functional effects of variants were predicted using the Ensembl database’s Variant Effect Predictor tool[54].

## Supporting Information

S1 Fig Bony deficiency in NSDTR individuals is largely restricted to the premaxillary bone. Rostral views of rendered reconstructed microCT scans of a normal (left) and affected (cleft #1; right) individual. Top row: full rendered view showing deficient premaxillary bone, with four anteriorly located incisors in the remaining ventral premaxillary segment. The lateral-most incisor on each side is positioned in a significantly more posterior position and without obvious alveolar bone supporting them. The nasal bones, maxillae and other cranial bones appear largely normal. Middle row: an anterior virtual coronal cutaway highlighting the near complete deficiency of premaxillary derived alveolar bone (compare green arrowheads), which is supported by the absence of the maxillary-premaxillary suture (compare yellow arrowheads). The main exception is the ventral premaxillary segment, which extends caudally to approximate with the vomer, similar to that seen in the normal individual. Bottom row: a more posterior virtual coronal cutaway showing deficient palatal bone (compare white arrowheads) and reduced ossification of the nasal conchae (white arrow). There appears to have been some compensatory expansion of the maxillae proper, with slightly widening of the skull at the level of the midface.
(TIF)

S2 Fig Bony and soft tissue deficiency in NSDTRs with cleft lip/palate. Ventral palatal views of two cleft individuals compared to an unaffected (normal) individual. The first cleft individual (cleft #1) is the same individual as that shown in S1 Fig. In contrast to the cleft #1, the second individual with cleft lip/palate (cleft #2) shows one palatal shelf having partially fused with the nasal septum. Consistent with this partial anterior fusion, the prolabium in this individual is not as anterior pronounced as that in cleft #1. The palatal rugae in both cleft individuals appear to have developed normally. The palatal deficiency (bony and soft tissue) may be mostly secondary to the premaxillary deficiency. Note the canines and molars in the cleft #1 individual appear unaffected. The central four incisors are small and malformed, while the two lateral incisors are large but abnormally positioned.
(TIF)

S3 Fig GWAS results for CL/P in Guatemalans. Analysis included 125 affected and 812 unaffected participants. A. Q-Q plot of SNP-wise p-values from DFAM analysis indicates no genomic inflation. B. Q-Q plot of gene-wise p-values from VEGAS analysis indicates no genomic inflation.
(DOCX)

S4 Fig Meta-analysis (-log10-transformed p-values) of the Guatemalan *ADAMTS20* results with those from Caucasian trios. Trios from Beaty et al (1). Stouffer’s method was used to combine p-values.
(DOCX)

S5 Fig CL/P Pedigrees with novel ADAMTS20 missense variants identified in human cases. A. Pedigree of a Filipino family with nonsyndromic CL/P. Both affected siblings inherited two novel missense variants, V586A and K601R from the unaffected mother. B. Pedigree of a Filipino family with nonsyndromic CL/P. The novel variant, Q1353H, was found in the proband and unaffected father and sibling. Note: No var.- variant allele not present; Not seq.- sample unavailable for sequencing. C. Pedigree of a syndromic CL/P proband with CL/P, facial asymmetry, and a single transverse palmar crease. The variant, p.A1108T, did not segregate with CL/P in the family.
(DOCX)

S6 Fig Population structure of the Guatemalan study subjects. For analyses of the population structure of the Guatemalan study population we used principal component analysis (PCA) implemented in R package “SNPRelate”. To select SNPs for PCA, we started from a pool of autosomal SNPs from the GWAS with missing call rate <5% and minor allele frequency >5% across all study subjects. There are 490,120 such SNPs. Then we performed linkage disequilibrium pruning using SNPRelate by recursively removing SNPs within a sliding window of 5 Mb so that no pairs of study subjects had a genotypic correlation r>0.45. The resulting 113,686 SNPs were used to generate the principal components. Shown is a plot of the first two eigenvectors from an analysis of 544 unrelated study subjects, along with 1,201 unrelated HapMap III controls (CEU, YRI, CHB, JPT, CHD, MKK, ASW, GIH, MEX, TSI, and LWK). The first eigenvector, accounting for 8.12% of the variance, separates the self-identified Guatemalan and White subjects from the self-identified African and African American subjects. The second eigenvector, accounting for 3.67% of the variance, separates the self-identified Guatemalan from the White and Asian subjects. Note also the partial overlap between the Guatemalan subjects and the HapMap MEX subjects, reflecting the similar ethnic backgrounds between the populations.
(DOCX)

S1 Table Summary of dogs that were whole genome sequenced. (DOCX)

S2 Table Summary of variant effects that segregated across the 33 dog genomes. (DOCX)

S3 Table Summary of allele frequencies of canine genotyping. (DOCX)

S4 Table Human GWAS SNPs with DFAM p-value less than 0.0001. (DOCX)

S5 Table Variants found in nonsyndromic CL/P cases from Guatemala and the Philippines. (DOCX)

S6 Table Primer sequences and annealing temperatures for Canine sequencing. (DOCX)

S7 Table Primer sequences and annealing temperatures for Human Sanger sequencing. (DOCX)
