# Supplementary figures and images for: Genome-Wide Association Studies in Dogs and Humans Identify ADAMTS20 as a Risk Variant for Cleft Lip and Palate
Source: PLoS Genet. 2015 Mar 23;11(3):e1005059. doi: 10.1371/journal.pgen.1005059 (PMC4370697; doi:10.1371/journal.pgen.1005059)

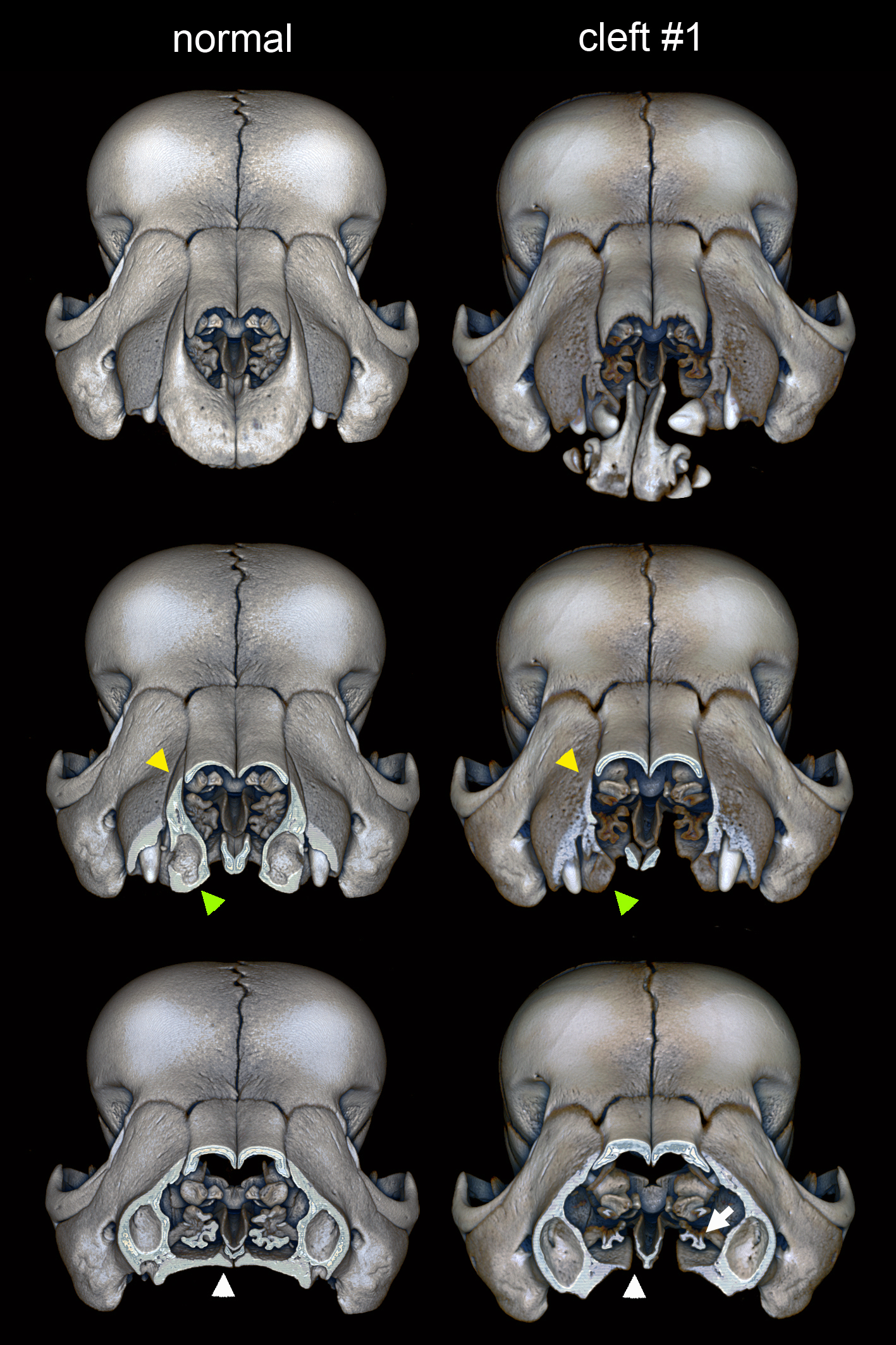

Supplement: S1 Fig — Rostral views of rendered reconstructed microCT scans of a normal (left) and affected (cleft #1; right) individual. Top row: full rendered view showing deficient premaxillary bone, with four anteriorly located incisors in the remaining ventral premaxillary segment. The lateral-most incisor on each side is positioned in a significantly more posterior position and without obvious alveolar bone supporting them. The nasal bones, maxillae and other cranial bones appear largely normal. Middle row: an anterior virtual coronal cutaway highlighting the near complete deficiency of premaxillary derived alveolar bone (compare green arrowheads), which is supported by the absence of the maxillary-premaxillary suture (compare yellow arrowheads). The main exception is the ventral premaxillary segment, which extends caudally to approximate with the vomer, similar to that seen in the normal individual. Bottom row: a more posterior virtual coronal cutaway showing deficient palatal bone (compare white arrowheads) and reduced ossification of the nasal conchae (white arrow). There appears to have been some compensatory expansion of the maxillae proper, with slightly widening of the skull at the level of the midface. (TIF) [file pgen.1005059.s001.tif]

**Figure S3. GWAS results for CL/P in Guatemalans**


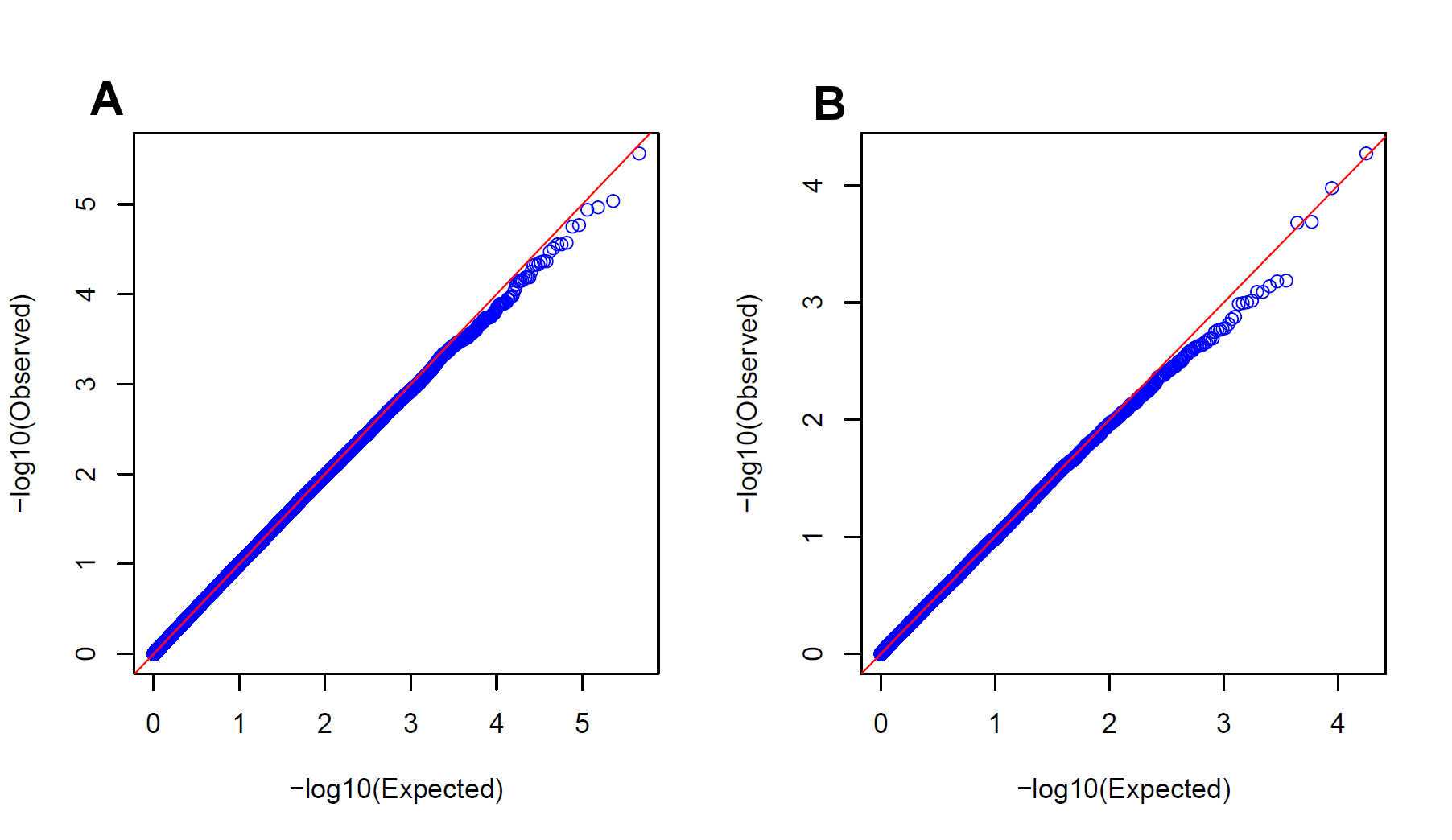

Supplement: S3 Fig — Analysis included 125 affected and 812 unaffected participants. A. Q-Q plot of SNP-wise p-values from DFAM analysis indicates no genomic inflation. B. Q-Q plot of gene-wise p-values from VEGAS analysis indicates no genomic inflation. (DOCX) [file pgen.1005059.s003.docx]

**Figure S5. CL/P Pedigrees with novel *ADAMTS20* missense variants identified in human cases**

**
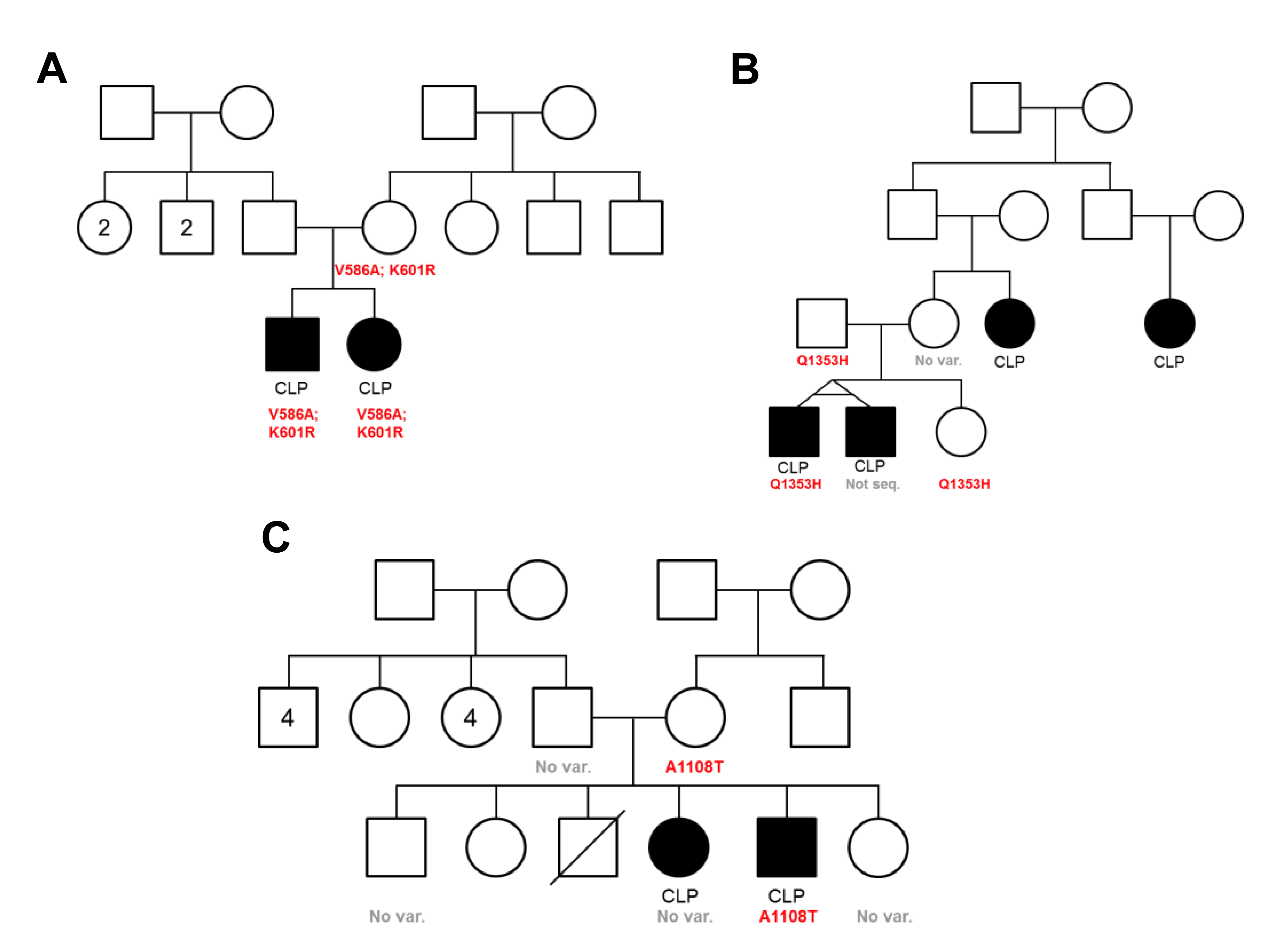
**

Supplement: S5 Fig — A. Pedigree of a Filipino family with nonsyndromic CL/P. Both affected siblings inherited two novel missense variants, V586A and K601R from the unaffected mother. B. Pedigree of a Filipino family with nonsyndromic CL/P. The novel variant, Q1353H, was found in the proband and unaffected father and sibling. Note: No var.- variant allele not present; Not seq.- sample unavailable for sequencing. C. Pedigree of a syndromic CL/P proband with CL/P, facial asymmetry, and a single transverse palmar crease. The variant, p.A1108T, did not segregate with CL/P in the family. (DOCX) [file pgen.1005059.s005.docx]

**Figure S6**. **Population structure of the Guatemalan study subjects**.


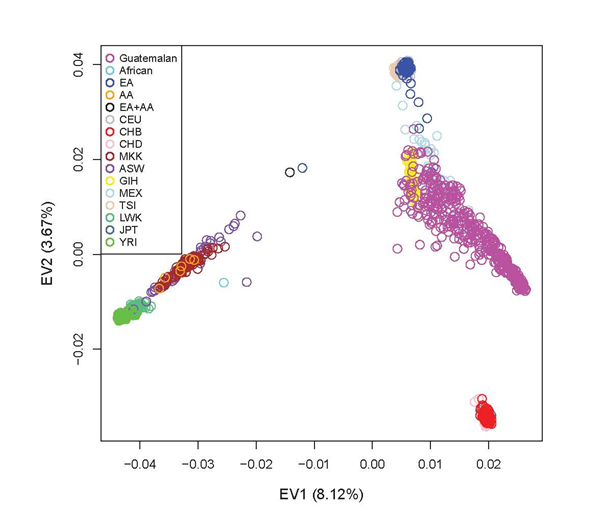

Supplement: S6 Fig — For analyses of the population structure of the Guatemalan study population we used principal component analysis (PCA) implemented in R package “SNPRelate”. To select SNPs for PCA, we started from a pool of autosomal SNPs from the GWAS with missing call rate <5% and minor allele frequency >5% across all study subjects. There are 490,120 such SNPs. Then we performed linkage disequilibrium pruning using SNPRelate by recursively removing SNPs within a sliding window of 5 Mb so that no pairs of study subjects had a genotypic correlation r>0.45. The resulting 113,686 SNPs were used to generate the principal components. Shown is a plot of the first two eigenvectors from an analysis of 544 unrelated study subjects, along with 1,201 unrelated HapMap III controls (CEU, YRI, CHB, JPT, CHD, MKK, ASW, GIH, MEX, TSI, and LWK). The first eigenvector, accounting for 8.12% of the variance, separates the self-identified Guatemalan and White subjects from the self-identified African and African American subjects. The second eigenvector, accounting for 3.67% of the variance, separates the self-identified Guatemalan from the White and Asian subjects. Note also the partial overlap between the Guatemalan subjects and the HapMap MEX subjects, reflecting the similar ethnic backgrounds between the populations. (DOCX) [file pgen.1005059.s006.docx]
