# Supplementary material for: Genome-Wide Association Studies in Dogs and Humans Identify ADAMTS20 as a Risk Variant for Cleft Lip and Palate
Source: PLoS Genet. 2015 Mar 23;11(3):e1005059. doi: 10.1371/journal.pgen.1005059 (PMC4370697; doi:10.1371/journal.pgen.1005059)
Supplement: S4 Fig — Trios from Beaty et al (1). Stouffer’s method was used to combine p-values. (DOCX) [file pgen.1005059.s004.docx]

**
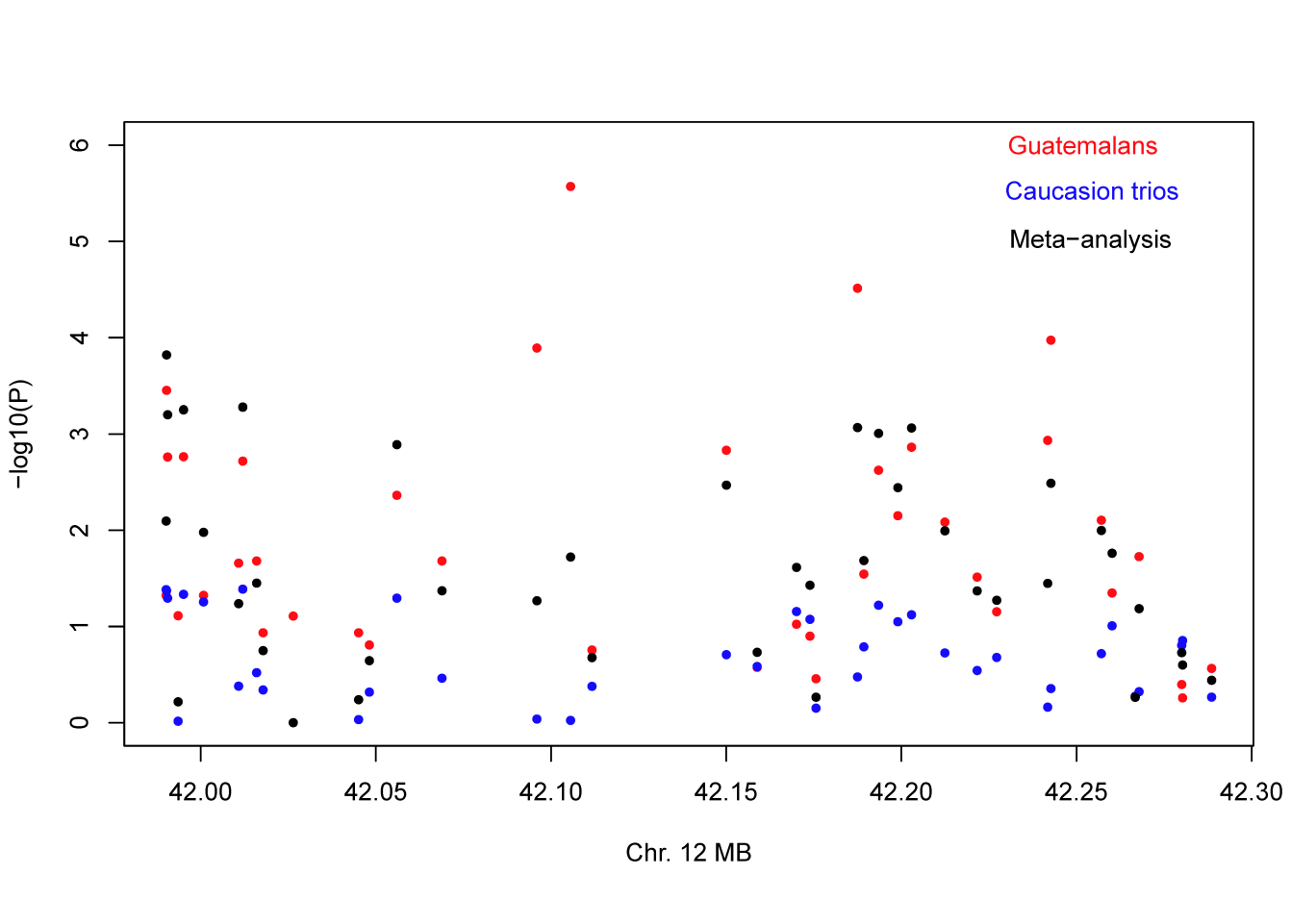
Figure S4. Meta-analysis (-log10-transformed p-values) of the Guatemalan *ADAMTS20* with those from Caucasian trios**

Trios from Beaty et al [1].

1. Beaty TH, Murray JC, Marazita ML, Munger RG, Ruczinski I, et al. (2010) A genome-wide association study of cleft lip with and without cleft palate identifies risk variants near MAFB and ABCA4. Nat Genet 42: 525-529.
