# Supplementary material for: Genome-Wide Association Studies in Dogs and Humans Identify ADAMTS20 as a Risk Variant for Cleft Lip and Palate
Source: PLoS Genet. 2015 Mar 23;11(3):e1005059. doi: 10.1371/journal.pgen.1005059 (PMC4370697; doi:10.1371/journal.pgen.1005059)
Supplement: S1 Table — (DOCX) [file pgen.1005059.s007.docx]

| Breed | Number of Dogs |
| --- | --- |
| Bearded Collies | 4 |
| Dalmatians | 6 |
| Kelpies | 7 |
| Miniature Schnauzers | 2 |
| Nova Scotia Duck Tolling Retrievers | 8 |
| Pugs | 4 |
| Weimaraners | 2 |
| Total | 33 |

**Table S1. Summary of dogs that were whole genome sequenced**
