# Supplementary material for: Genome-Wide Association Studies in Dogs and Humans Identify ADAMTS20 as a Risk Variant for Cleft Lip and Palate
Source: PLoS Genet. 2015 Mar 23;11(3):e1005059. doi: 10.1371/journal.pgen.1005059 (PMC4370697; doi:10.1371/journal.pgen.1005059)
Supplement: S2 Table — (DOCX) [file pgen.1005059.s008.docx]

**Table S2. Summary of variant effects that segregated across the 33 dog genomes**

| Variant Effect | Number of Variants |
| --- | --- |
| Intron | 71 |
| Intergenic | 58 |
| Upstream | 10 |
| Downstream | 1 |
| Synonymous  PUS7L:c.278A>G *p.(=)* | 1 |
| Frameshift  *ADAMTS20*: c.1360_1361delAA p.Lys453Ilefs*3 | 1 |
| Total | 142 |

All variant effects were predicted by SnpEff software [[1]](#_ENREF_2).

1. Cingolani P, Platts A, Wang le L, Coon M, Nguyen T, et al. (2012) A program for annotating and predicting the effects of single nucleotide polymorphisms, SnpEff: SNPs in the genome of Drosophila melanogaster strain w1118; iso-2; iso-3. Fly (Austin) 6: 80-92.
