# Supplementary material for: Genome-Wide Association Studies in Dogs and Humans Identify ADAMTS20 as a Risk Variant for Cleft Lip and Palate
Source: PLoS Genet. 2015 Mar 23;11(3):e1005059. doi: 10.1371/journal.pgen.1005059 (PMC4370697; doi:10.1371/journal.pgen.1005059)
Supplement: S3 Table — (DOCX) [file pgen.1005059.s009.docx]

**Table S3. Summary of allele frequencies of canine genotyping.**

| Sample | N | Allele Frequency* |
| --- | --- | --- |
| CLPS NSDTRs | 13 | 100% |
| NSDTRs with CL and/or CP | 34 | 44% |
| NSDTRs without CL and/or CP | 97 | 3% |
| Non-NSDTRs without CL and/or CP | 288 | 0% |
| Non-NSDTRs with CL and/or CP | 53 | 0% |

*Indicates dogs that have at least one copy of the deletion.
