# Supplementary material for: Genome-Wide Association Studies in Dogs and Humans Identify ADAMTS20 as a Risk Variant for Cleft Lip and Palate
Source: PLoS Genet. 2015 Mar 23;11(3):e1005059. doi: 10.1371/journal.pgen.1005059 (PMC4370697; doi:10.1371/journal.pgen.1005059)
Supplement: S4 Table — (DOCX) [file pgen.1005059.s010.docx]

**Table S4. Human GWAS SNPs with DFAM p-value less than 0.0001**

| CHR | BP | SNP | Major allele | Minor allele | OBS | EXP | CHISQ | P | GENE(S) |
| --- | --- | --- | --- | --- | --- | --- | --- | --- | --- |
| 1 | 187997774 | rs16831401 | C | A | 40 | 58.99 | 16.69 | 4.41E-05 | (none) |
| 1 | 238709790 | rs11578184 | C | A | 90 | 71.49 | 16.58 | 4.67E-05 | FMN2, GREM2 |
| 2 | 135462192 | rs10928526 | A | G | 51 | 35.4 | 17.55 | 2.79E-05 | MAP3K19 |
| 2 | 170476461 | rs7593011 | G | A | 58 | 42.55 | 15.22 | 9.56E-05 | UBR3 |
| 2 | 214370820 | rs6720110 | G | A | 70 | 53.26 | 15.79 | 7.08E-05 | SPAG16 |
| 3 | 156346113 | rs6801319 | G | A | 33 | 20.54 | 16.6 | 4.62E-05 | MME |
| 3 | 188969325 | rs12487326 | G | A | 55 | 40.46 | 15.97 | 6.44E-05 | BCL6 |
| 4 | 6452003 | rs7671165 | G | A | 42 | 28.29 | 16.73 | 4.31E-05 | PPP2R2C |
| 4 | 6452911 | rs7437478 | A | G | 42 | 28.29 | 16.73 | 4.31E-05 | PPP2R2C |
| 4 | 6459624 | rs12642310 | A | C | 39 | 26.22 | 15.95 | 6.50E-05 | PPP2R2C |
| 4 | 6460449 | rs16838740 | G | A | 39 | 26.21 | 15.96 | 6.46E-05 | PPP2R2C |
| 6 | 2947322 | rs4149353 | G | C | 16 | 29.66 | 16.54 | 4.76E-05 | NQO2 |
| 9 | 116070403 | rs2636860 | G | A | 17 | 30.44 | 15.79 | 7.06E-05 | COL27A1 |
| 10 | 7892634 | rs7904875 | A | G | 35 | 51.83 | 15.88 | 6.74E-05 | ATP5C1, TAF3 |
| 12 | 42105565 | rs10785430 | G | A | 45 | 64.41 | 22.03 | 2.69E-06 | ADAMTS20 |
| 12 | 42187460 | rs1510522 | C | A | 39 | 56.57 | 17.37 | 3.07E-05 | ADAMTS20 |
| 13 | 73286088 | rs1324061 | A | G | 55 | 73.67 | 17.19 | 3.38E-05 | KLF12 |
| 13 | 73292415 | rs9530247 | G | A | 89 | 69.02 | 19.68 | 9.15E-06 | KLF12 |
| 13 | 74229094 | rs11617463 | C | A | 23 | 13.18 | 15.78 | 7.12E-05 | LINC00347 |
| 13 | 74250287 | rs17063548 | G | A | 23 | 13.18 | 15.78 | 7.12E-05 | LINC00347 |
| 13 | 80196792 | rs17073177 | C | A | 56 | 39.82 | 18.48 | 1.72E-05 | (none) |
| 13 | 80229147 | rs7336234 | A | G | 56 | 39.85 | 18.41 | 1.78E-05 | (none) |
| 13 | 80288325 | rs7327912 | A | G | 57 | 40.35 | 19.23 | 1.16E-05 | (none) |
| 13 | 80380942 | rs1887077 | C | A | 57 | 40.9 | 17.65 | 2.66E-05 | (none) |
| 13 | 100779104 | rs1931084 | G | A | 51 | 35.72 | 15.36 | 8.89E-05 | NALCN |
| 14 | 95213400 | rs1743507 | A | C | 31 | 45.75 | 15.6 | 7.82E-05 | TCL6, TCL1B |
| 16 | 73827035 | rs3743613 | G | A | 85 | 65.36 | 19.38 | 1.07E-05 | BCAR1 |
| 18 | 59063280 | rs11872329 | G | A | 20 | 34.3 | 17.57 | 2.77E-05 | BCL2 |
| 22 | 22252983 | rs140174 | A | G | 91 | 74.55 | 16.22 | 5.63E-05 | IGLL1 |
