# Supplementary material for: Genome-Wide Association Studies in Dogs and Humans Identify ADAMTS20 as a Risk Variant for Cleft Lip and Palate
Source: PLoS Genet. 2015 Mar 23;11(3):e1005059. doi: 10.1371/journal.pgen.1005059 (PMC4370697; doi:10.1371/journal.pgen.1005059)
Supplement: S5 Table — (DOCX) [file pgen.1005059.s011.docx]

| **Table S5.** Variants found in nonsyndromic CL/P cases from Guatemala and the Philippines | | | | | | | | | | |
| --- | --- | --- | --- | --- | --- | --- | --- | --- | --- | --- |
| **Exon** | **Variant** | **Alleles** | **Guatemalan NSCL/P** | | | | **Filipino NSCL/P** | | | |
|  |  |  | AA | AB | BB | MAF | AA | AB | BB | MAF |
| **1** | rs143097007 | T/- | 18 | 2 | 0 | 5.0% | 14 | 5 | 0 | 13.2% |
|  | rs275602 | C/A | 16 | 1 | 0 | 2.9% | 14 | 0 | 0 | 0.0% |
|  | rs275601 | G/C | 16 | 1 | 0 | 2.9% | 14 | 0 | 0 | 0.0% |
| **3** | rs61925177 | A/C | 11 | 7 | 0 | 19.4% | 16 | 2 | 0 | 5.6% |
| **4** | rs78569367 | A/G | 17 | 1 | 0 | 2.8% | 19 | 0 | 0 | 0.0% |
| **7** | rs7297170 | C/A | 12 | 3 | 0 | 10.0% | 17 | 2 | 0 | 5.3% |
| **8** | rs2840127 | G/A | 5 | 6 | 2 | 38.5% | 0 | 4 | 14 | 88.9% |
| **9** | rs11182088 (P432P) | A/G | 4 | 8 | 3 | 46.7% | 0 | 4 | 14 | 88.9% |
|  | rs1461111978 | C/T | 15 | 1 | 0 | 3.1% | 18 | 0 | 0 | 0.0% |
|  | rs74913159 | C/T | 14 | 1 | 0 | 3.3% | 16 | 2 | 0 | 5.6% |
|  | rs4988573 | C/A | 8 | 6 | 2 | 31.3% | 16 | 2 | 0 | 5.6% |
|  | rs145981500 | -/TA | 13 | 3 | 0 | 9.4% | 15 | 3 | 0 | 8.3% |
| **12** | rs6582464 | T/C | 5 | 6 | 3 | 42.9% | 15 | 2 | 0 | 5.9% |
|  | rs11182086 | C/T | 9 | 5 | 0 | 17.9% | 2 | 12 | 3 | 52.9% |
|  | rs11182085 | A/G | 10 | 5 | 0 | 16.7% | 2 | 12 | 3 | 52.9% |
| **13-14** | chr12:43,846,511 | A/G | 13 | 0 | 0 | 0.0% | 15 | 2 | 0 | 5.9% |
|  | **chr12:43,846,472 (V596A)** | A/G | 13 | 0 | 0 | 0.0% | 16 | 1 | 0 | 2.9% |
|  | **chr12:43,846,457 (K601R)** | T/C | 13 | 0 | 0 | 0.0% | 16 | 1 | 0 | 2.9% |
|  | rs7307955 | T/C | 7 | 5 | 1 | 26.9% | 18 | 0 | 0 | 0.0% |
| **15** | rs61925148 | G/A | 11 | 7 | 0 | 19.4% | 17 | 2 | 0 | 5.3% |
|  | rs12307055 | G/A | 10 | 8 | 0 | 22.2% | 9 | 9 | 1 | 28.9% |
| **17-18** | rs3764469 | C/G | 14 | 2 | 0 | 6.3% | 16 | 2 | 0 | 5.6% |
| **19** | rs11428208 | T/- | 9 | 6 | 0 | 20.0% | 17 | 1 | 0 | 2.8% |
|  | rs7302446 (K876M) | T/A | 9 | 1 | 0 | 5.0% | 15 | 2 | 0 | 5.9% |
|  | rs7301900 | T/C | 9 | 1 | 0 | 5.0% | 16 | 2 | 0 | 5.6% |
| **22** | rs2201465 | G/T | 11 | 8 | 0 | 21.1% | 9 | 9 | 1 | 28.9% |
| **23** | rs11182071 | A/G | 5 | 5 | 3 | 42.3% | 0 | 5 | 14 | 86.8% |
|  | rs10880489 | G/T | 5 | 5 | 3 | 42.3% | 0 | 5 | 14 | 86.8% |
| **26** | rs11182068 | T/C | 6 | 0 | 0 | 0.0% | 2 | 0 | 5 | 71.4% |
| **27** | **chr12:43,821,159 (Q1353H)** | T/A | 11 | 0 | 0 | 0.0% | 18 | 1 | 0 | 2.6% |
|  | rs10161427 | G/A | 3 | 5 | 3 | 50.0% | 0 | 5 | 14 | 86.8% |
| **30-31** | rs17093250 | T/A | 6 | 9 | 0 | 30.0% | 2 | 9 | 6 | 61.8% |
| **36** | rs10880473 (R1784R) | A/G | 16 | 1 | 0 | 2.9% | 17 | 1 | 0 | 2.8% |
|  | rs10506226 (T1800T) | T/G | 15 | 1 | 0 | 3.1% | 17 | 1 | 0 | 2.8% |
| **37** | chr16:43,763,319 | G/T | 16 | 0 | 0 | 0.0% | 18 | 1 | 0 | 2.6% |
| **38** | chr16: 43,750,416 | A/G | 16 | 1 | 0 | 2.9% | 18 | 0 | 0 | 0.0% |
|  | |  |  |  |  |  |  |  |  |  |
