# Supplementary material for: Genome-Wide Association Studies in Dogs and Humans Identify ADAMTS20 as a Risk Variant for Cleft Lip and Palate
Source: PLoS Genet. 2015 Mar 23;11(3):e1005059. doi: 10.1371/journal.pgen.1005059 (PMC4370697; doi:10.1371/journal.pgen.1005059)
Supplement: S6 Table — (DOCX) [file pgen.1005059.s012.docx]

**Table S6.** Primer sequences and annealing temperatures for Canine sequencing.

| cDNA Sequencing | | | | |
| --- | --- | --- | --- | --- |
| Genomic location |  | Sequence | Product Size | Annealing Temperature |
| 10492526 -10493216 | F/R | TCCGGAGGCTGCTGAG/ GGAACACCTCGCCAAACTC | 300 | 58 |
|  | S | GCCGGGACTGCTGCT |  |  |
| 10493199-10493493 | F/R | GAGTTTGGCGAGGTGTTCC/ TCCGCAGAGGCTGAAGAC | 301 | 60 |
| 10493283-10528289 | F/R | CTACCGGATCAGCGCCTA/ CAGCTGTAACCATGACTTCCAC | 574 | 59 |
| 10528193- 10564701 | F/R | ATGGTTTTAGGGCACCCTTC/ AGTCTTGCTTGCCTTTTGGA | 1147 | 59 |
| 10563327-10574423 | F/R | AATGGAAACACGTCCGGTAG/ CTCTTCCACGGGGATATTGA | 879 | 59 |
| 10574174-10584757 | F/R | ACTGATCGGCTGGAAGAAGA/ CATTTAACAGCACGCATCTGA | 877 | 59 |
| 10583205-10587871 | F/R | GGTGCCAGCTGAATGAAGAT/ AGTGGCACTCTGTCCGTCTT | 880 | 59 |
| 10583289-10587083 | F/R | TGATCCCCATGGTCCTGTTC/ CTTTGAGACCATGTGAGCTTCA | 742 | 59 |
| 10586902-10626861 | F/R | GCAGTTCGCCCTTTGATAGA/ AAGGCAGCACAGGACATTCT | 1179 | 59.5 |
| 10624005 - 10651142 | F/R | GGGTGGCTGAAGAAATGTGT/ TATCTTCATCCCCGTTCCTG | 1066 | 59 |
| 10634945-10654236 | F/R | ATTTGCCACAGCTGGAGATT/ AATGAGCACCGGTTATTTGC | 305 | 59 |
| 38469097-38470100 (GAPDH) | F/R | AAGATTGTCAGCAATGCCTCC/ CCAGGAAATGAGCTTGACAAA | 507 | 58 |
| Genotyping | | | | |
| 10553454-10553588 | F/R | TGGACCTGGTCAAAATGTAGTC/  TGAACCACTTTAAAAGAACAAATGA | wild-type: 135  mutant: 133 | 58 |
| Real-Time PCR | | | | |
| 10543811-10551461 | F/R | GGAAGACATTTGTGGAGCTAGAG/  GCGCTGAGTCCATTTTCTT | 100 | 60 |

Genomic locations are based on the CanFam3.1 assembly and refer to chromosome 27 base pair locations. Primers were designed to cover cDNA sequence of *ADAMTS20*. F – forward primer. R– Reverse primer. S – primer used for sequencing. All primers are in a 3’-5’ orientation. Expression of *ADAMTS20* was evaluated in day 30 embryos, unaffected adult beagle tissue from cerebellum, cerebral cortex, heart, kidney, liver, skeletal muscle, skin, spinal cord, spleen, testis, and thymus. GAPDH was amplified in these tissues to ensure that equivalent amounts of cDNA were added.
