# Supplementary material for: Genome-Wide Association Studies in Dogs and Humans Identify ADAMTS20 as a Risk Variant for Cleft Lip and Palate
Source: PLoS Genet. 2015 Mar 23;11(3):e1005059. doi: 10.1371/journal.pgen.1005059 (PMC4370697; doi:10.1371/journal.pgen.1005059)
Supplement: S7 Table — (DOCX) [file pgen.1005059.s013.docx]

**Table S7. Primer sequences and annealing temperatures for Human Sanger sequencing.**

| **EXON** | **Size** | **Annealing Temperature** | **Primer Sequence (5'-3')** | |
| --- | --- | --- | --- | --- |
| 1 | 659 | 63 | F | GCTGAACCTGAGCAGAGAGTTT |
|  |  |  | R | CTTCCCCAGTACCTCCTAACCT |
| 2 | 707 | 64 | F | TAGTGGTAGGGTCTTTGCTTGG |
|  |  |  | R | TGAACTCCAGGGTCATCAGC |
| 3 | 400 | 63 | F | GGCGACTGTTAGGAAAAATTCA |
|  |  |  | R | AAGCCATTCCACAATGTATGC |
| 4 | 657 | 57 | F | TTCTTTTATTTTCATGTTGGGAAC |
|  |  |  | R | CAGAAGCCATTCATGTGAGAAA |
| 5-6 | 864 | 63.5 | F | TCTGTGACTATTTCAACTTACTTTTGA |
|  |  |  | R | AAAGGATTGTATGGGAAGGGTTA |
| 7 | 390 | 55 | F | GCTTATTGATGTGGACAAATGG |
|  |  |  | R | AATTTACAGCAGCAGAATGAAATA |
| 8 | 632 | 60 | F | GCTTTTCTATTACCAATCAACACA |
|  |  |  | R | GAGCCAGAGTTAGCATTTCAGG |
| 9 | 632 | 56 | F | TGCCAGATCACCAGCCTATAAT |
|  |  |  | R | TTCATCACAAGAAAGAATCCAGAA |
| 10 | 437 | 55 | F | TGATATTGATGTGAGTTGGTTTTT |
|  |  |  | R | TTCCTTTTCTTAACCTCTTTTTGA |
| 11 | 569 | 60 | F | ATTTCCACTCCAATTCATAACTCC |
|  |  |  | R | TATTCATCTGCCATAGCTTAGAAA |
| 12 | 735 | 63.5 | F | CTCCCCAATGACACTCTAAATCTT |
|  |  |  | R | CATAAGTGAAAGTAGCCAGGATGTT |
| 13-14 | 660 | 57 | F | GTGGGACTTCACTGGTTTTCTA |
|  |  |  | R | AGGTTGCTTTGTTGGAATGC |
| 15 | 451 | 51 | F | ATTGATTGTAATTGTGTCTTGAAG |
|  |  |  | R | TTAATGGGAGGATTATTCACATATC |
| 16 | 363 | 55 | F | CAACAACCACTTTTTCTCAATCC |
|  |  |  | R | TTCACATCAACTACAGACTTCCA |
| 17-18 | 604 | 59 | F | ATTGTTGATTGGTGTTACTATTTCAT |
|  |  |  | R | GTATGGACAGTGGGGAGGATT |
| 19 | 779 | 56 | F | AATGCCTGTCCCATCTTCTGTA |
|  |  |  | R | GAATGTCAAGGAAATCAATCAAT |
| 20-21 | 777 | 64 | F | TTTCAGAGGTTTTTGAGGAAGC |
|  |  |  | R | TTTAATAGTTCCAAGCATCATGTGT |
| 22 | 508 | 56 | F | AATGGATGTTTCAGTGGGACTT |
|  |  |  | R | ATTTCAGGGTTGCTGTTGTTTT |
| 23 | 643 | 61 | F | TGCTGTTCCTTCTTTGTCTCTTT |
|  |  |  | R | GCCTCTCTCTCACACATACACG |
| 24 | 511 | 55 | F | CTTGAGACATACAGAAGAAGTGAAGT |
|  |  |  | R | CAAATAAAAGCCGATACAGCCTA |
| 25-26 | 953 | 58 | F | CAGCTATGTAAAAGGGACATGG |
|  |  |  | R | AATTTTGGGATTTAGGTTTATTCA |
| 27 | 734 | 55 | F | TGTGATTCCAATGAAGGAGATTC |
|  |  |  | R | TGAGTGACCTAAACCATGACAGA |
| 28 | 285 | 57 | F | AAACCACAGTTTTGAATTTTCC |
|  |  |  | R | CAATGAACTCTCCCTCACTCAG |
| 29 | 442 | 61 | F | GGTCCAGAGCAGATTGGTAACT |
|  |  |  | R | TTTAGAGAAGCAAAACATTCAAGG |
| 30-31 | 800 | 58 | F | TTGATTCGCTTGTCAGAGTTTC |
|  |  |  | R | TGTCAGTGGTCTCATTCCTTAGA |
| 32 | 596 | 50 | F | AATGTTTTATTCCTCCAATGCT |
|  |  |  | R | ATTACCAGATTTCCCACACCTA |
| 33-35 | 886 | 56 | F | CTCAGTGTGCTCCAGAAATACC |
|  |  |  | R | TCTGAACAGGCATTAACAACATC |
| 36 | 600 | 60 | F | TCGGACATAGAGAAGGGAAGAA |
|  |  |  | R | TGTCATTGCAGAGGAAGTATG |
| 37 | 443 | 61 | F | CCCTTTGTTAGGGCAATTTATG |
|  |  |  | R | TGTGAAGTTTTGCATTTCTATTTACTT |
| 38 | 800 | 61 | F | AATGAAGGGTCAAAAATGGAAC |
|  |  |  | R | CAGTGAGGGAAGAAAACAAAAA |
| 39 | 593 | 63.5 | F | TAACTTTCCCATGTCCACCAAT |
|  |  |  | R | GTCCAGGCCAAGATGTTAAGAG |
